# Glow-in-the-Dark Infectious Disease Diagnostics Using CRISPR-Cas9-Based Split Luciferase Complementation

**DOI:** 10.1021/acscentsci.2c01467

**Published:** 2023-03-15

**Authors:** Harmen J. van der Veer, Eva A. van Aalen, Claire M. S. Michielsen, Eva T. L. Hanckmann, Jeroen Deckers, Marcel M. G. J. van Borren, Jacky Flipse, Anne J. M. Loonen, Joost P. H. Schoeber, Maarten Merkx

**Affiliations:** † Laboratory of Chemical Biology, Department of Biomedical Engineering, Eindhoven University of Technology, P.O. Box 513, Eindhoven 5600 MB, The Netherlands; ‡ Institute for Complex Molecular Systems, Eindhoven University of Technology, P.O. Box 513, Eindhoven 5600 MB, The Netherlands; § Department of Clinical Chemistry, Rijnstate Hospital, P.O. Box 9555, Arnhem 6800 TA, The Netherlands; ∥ Laboratory for Medical Microbiology and Immunology, Rijnstate Hospital, P.O. Box 8, Velp 6880 AA, The Netherlands; ⊥ Research Group Applied Natural Sciences, Fontys University of Applied Sciences, Eindhoven 5612 AP, The Netherlands; # Pathologie-DNA, Lab for Molecular Diagnostics, Location Jeroen Bosch Hospital, ’s-Hertogenbosch 5223 GZ, The Netherlands

## Abstract

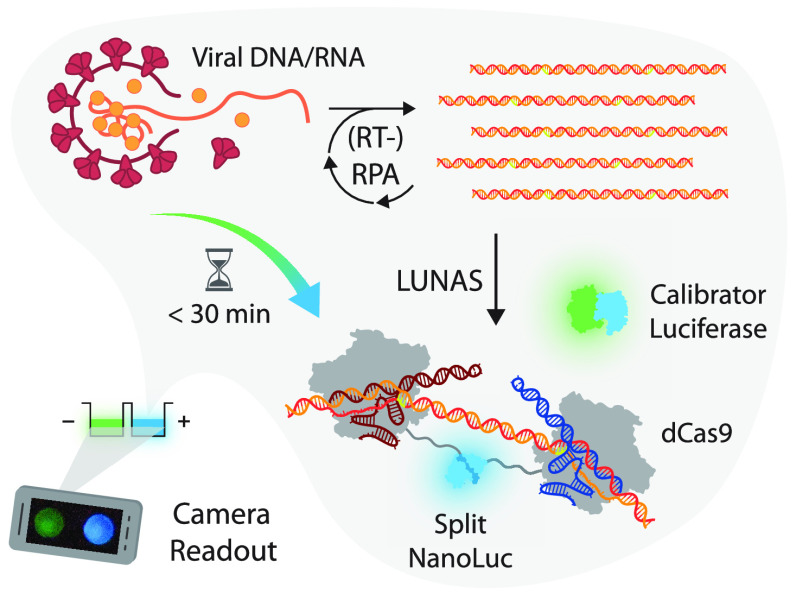

Nucleic acid detection methods based on CRISPR and isothermal amplification techniques show great potential for point-of-care diagnostic applications. However, most current methods rely on fluorescent or lateral flow assay readout, requiring external excitation or postamplification reaction transfer. Here, we developed a bioluminescent nucleic acid sensor (LUNAS) platform in which target dsDNA is sequence-specifically detected by a pair of dCas9-based probes mediating split NanoLuc luciferase complementation. LUNAS is easily integrated with recombinase polymerase amplification (RPA), providing attomolar sensitivity in a rapid one-pot assay. A calibrator luciferase is included for a robust ratiometric readout, enabling real-time monitoring of the RPA reaction using a simple digital camera. We designed an RT-RPA-LUNAS assay that allows SARS-CoV-2 RNA detection without the need for cumbersome RNA isolation and demonstrated its diagnostic performance for COVID-19 patient nasopharyngeal swab samples. Detection of SARS-CoV-2 from samples with viral RNA loads of ∼200 cp/μL was achieved within ∼20 min, showing that RPA-LUNAS is attractive for point-of-care infectious disease testing.

## Abstract 2

The glow-in-the-dark RPA-LUNAS assay combining isothermal amplification and CRISPR-mediated luciferase activation enables sensitive camera-based detection of pathogen DNA/RNA within 20 min.

## Introduction

Identification of pathogens by the detection of their nucleic acid fingerprints is a key strategy in clinical diagnostics, biomedical research, and food and environmental safety monitoring. The widely used quantitative polymerase chain reaction (qPCR) is highly sensitive but requires expensive thermal cycling equipment and expert technicians, restricting its use to centralized laboratories that process samples sent in from local collection facilities. The resulting long time from sample to result and the limited diagnostic access in low-resource areas have stimulated the development of rapid, portable, and easy-to-use nucleic acid diagnostics that can be deployed at the point-of-care (POC).^1,2^

Unlike PCR, isothermal nucleic acid amplification methods such as loop-mediated isothermal amplification (LAMP) and recombinase polymerase amplification (RPA) do not require thermal cycling for exponential accumulation of amplicons to detectable levels and hence have gained interest for development of POC diagnostic tests.^3−6^ Both LAMP and RPA are very rapid and highly sensitive techniques that operate at constant temperatures of ∼65 °C and ∼40 °C, respectively. Although such low temperatures ease some requirements for equipment, they come with the risk of nonspecific amplification, leading to potential spurious results when using nonspecific detection methods based on pH-change or fluorescent detection of total dsDNA. Therefore, various sequence-specific detection strategies have been developed for stringent target detection, including recent CRISPR diagnostic methods (CRISPR-Dx), which are mostly based on the collateral cleavage activity of type V (Cas12) and VI (Cas13) CRISPR effector proteins.^7−10^ Following RPA- or LAMP-based target preamplification, Cas12 enzymes complexed with a short guide RNA (crRNA) bind sequence specifically to the amplicons, whereas Cas13 ribonucleoproteins (RNPs) require an additional in vitro transcription (IVT) step for converting dsDNA amplicons to target RNA.^7,8^ Target binding in turn triggers nonspecific nuclease activity, which is detected using cleavable fluorophore-quencher reporter nucleic acids.^7,8,11^ However, the external excitation required for fluorescent detection gives rise to autofluorescence and scattering, limiting the sensitivity especially in complex media such as blood plasma.^12^ Alternatively, the cleavage of reporter molecules can be visualized in a Laminar Flow Assay (LFA), which can be performed using simple paper-based devices and can be read by eye. However, LFA is inherently nonquantitative and prone to cross contamination due to postamplification reaction transfer.^11,13^ Since the beginning of the COVID-19 pandemic, several methods based on these principles have been developed for the detection of SARS-CoV-2, demonstrating sensitive detection from COVID-19 patient nasopharyngeal or saliva samples and featuring a sample-to-answer time of typically less than an hour.^14−20^ In contrast to rapid antigen tests based on lateral flow immunoassays that are now widely available for COVID-19 diagnosis, these isothermal nucleic acid tests are generally more sensitive but involve more complex test devices and procedures.^21,22^

Bioluminescent sensors require no external excitation and hence do not suffer from the sources of noise typical of fluorescence-based methods in complex samples. Bioluminescence also does not require sophisticated equipment but can be detected with any ordinary digital camera, making this type of readout particularly attractive for point-of-care applications.^23−25^ Our group has previously reported the use of bioluminescence for detection of ssDNA and ssRNA targets through a luciferase conjugate molecular beacon, and a similar bioluminescent sensor concept based on toehold-mediated strand exchange was recently described by Winssinger and co-workers.^26,27^ However, these methods are limited to detecting single stranded nucleic acids at picomolar levels, whereas attomolar sensitivity is typically required in diagnostics.^28−30^ A bioluminescent dsDNA sensor based on dCas9-mediated split firefly luciferase complementation has been reported by Zhang and co-workers but with a relatively modest limit of detection of ∼50 pM.^31^

**Figure 1:**
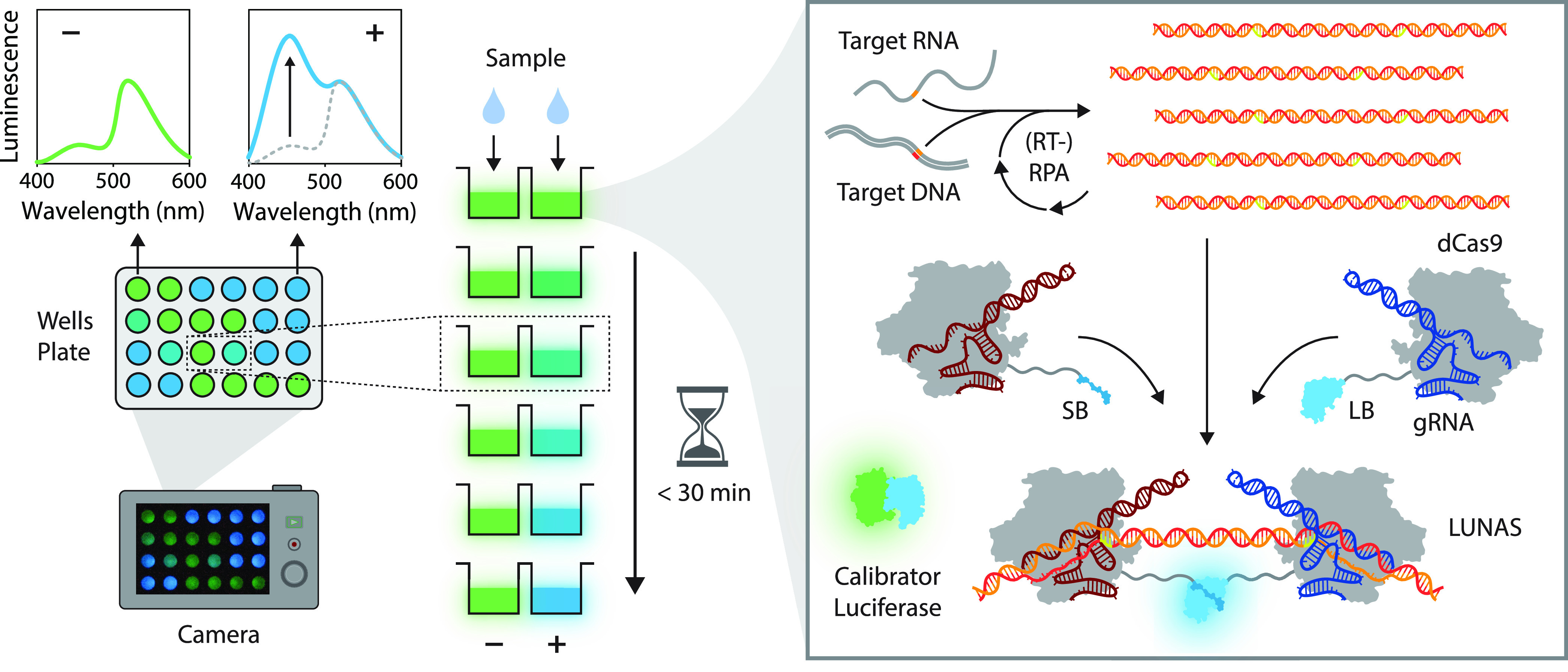
Schematic overview of the (RT-)RPA-LUNAS method. The presence of the target nucleic acid in a sample results in rapid exponential amplification of the target region through (RT-)RPA at a constant temperature of 40 °C. Two dCas9 proteins, fused to small BiT (SB) and large BiT (LB) split NanoLuc fragments, respectively, and complexed with distinct guide RNAs (gRNA), bind to the resulting amplicons in close proximity of each other, allowing complementation of SB and LB and a resulting increase in blue luminescence (*λ*_max_ ∼ 460 nm) upon oxidation of the furimazine substrate. An mNeonGreen-NanoLuc (mNG-NL) fusion protein is included as a calibrator luciferase to correct for substrate turnover, emitting green light (*λ*_max_ ∼ 520 nm) through Bioluminescence Resonance Energy Transfer (BRET). All components are combined in a one-pot reaction and, following the addition of a sample, the luminescence can be recorded by a digital camera. In the absence of target nucleic acid, no blue-light-emitting LUNAS complexes form, and mostly green luminescence is detected (low blue/green ratio), whereas the presence of the target nucleic acid results in an increase in blue signal (blue/green ratio high) within ∼30 min.

Here we report LUNAS (Luminescent Nucleic Acid Sensor), a highly improved generic platform for sequence specific, bioluminescent dsDNA detection based on dCas9-mediated split NanoLuc complementation, that can be easily combined with RPA isothermal amplification for rapid one-pot real-time amplification and detection of nucleic acids (Figure 1). In the RPA-LUNAS method, low input concentrations of target are efficiently amplified by (RT-)RPA, and the accumulating dsDNA amplicons serve as templates for recruitment of dCas9-LargeBiT and dCas9-SmallBiT ribonucleoproteins (RNPs) in close proximity, allowing reconstitution of the bright, blue light-emitting NanoLuc luciferase.^32^ Addition of a green light-emitting calibrator luciferase previously developed in our group provides a robust blue-over-green ratiometric output.^25,46^ RPA-LUNAS can be readily adapted to new targets, both DNA and RNA, by designing matching RPA primers and guide RNAs. Here, we demonstrate the performance of RT-RPA-LUNAS in SARS-CoV-2 detection, allowing direct detection of SARS-CoV-2 RNA in saliva, viral transport medium (VTM), and liquid amies medium (eSwab) without extraction. Finally, we demonstrate the detection of SARS-CoV-2 virus in COVID-19 patient samples within 10–30 min using an ordinary digital camera for readout, illustrating the potential for point-of-care application.

## Results

### LUNAS Design and Characterization

Prior to the advent of collateral cleavage based CRISPR diagnostics, Zhang and co-workers reported a bioluminescent dsDNA sensor based on dCas9-mediated split firefly luciferase complementation featuring a sensitivity of ∼50 pM.^31^ We took this concept as a starting point for the design of a more sensitive sensor employing the brighter, smaller, and more stable split NanoLuc luciferase.^32^ In our design, the 1.3 kDa small BiT (SB; *K*_D_ = 2.5 μM) and 18.1 kDa large BiT (LB) luciferase fragments are fused to the C-terminus of *S. pyogenes* dCas9 via a flexible peptide linker (see the Supporting Information for the sequence). Complexation of the two dCas9 fusion proteins with distinct guide RNAs (gRNAs A & B) that are complementary to adjacent protospacer sequences in the target DNA enables the binding of the sensor RNPs to the DNA in close proximity of each other, resulting in the reconstitution of the luciferase. As gRNA exchange is known not to occur in vitro, separate incubation of dCas9-SB with gRNA_A and dCas9-LB with gRNA_B to form the sensor RNPs prior to the DNA sensing reaction avoids obtaining a statistical mixture of RNP variants wherein half of the resulting RNP:DNA complexes would not result in luciferase reconstitution.^33^

**Figure 2:**
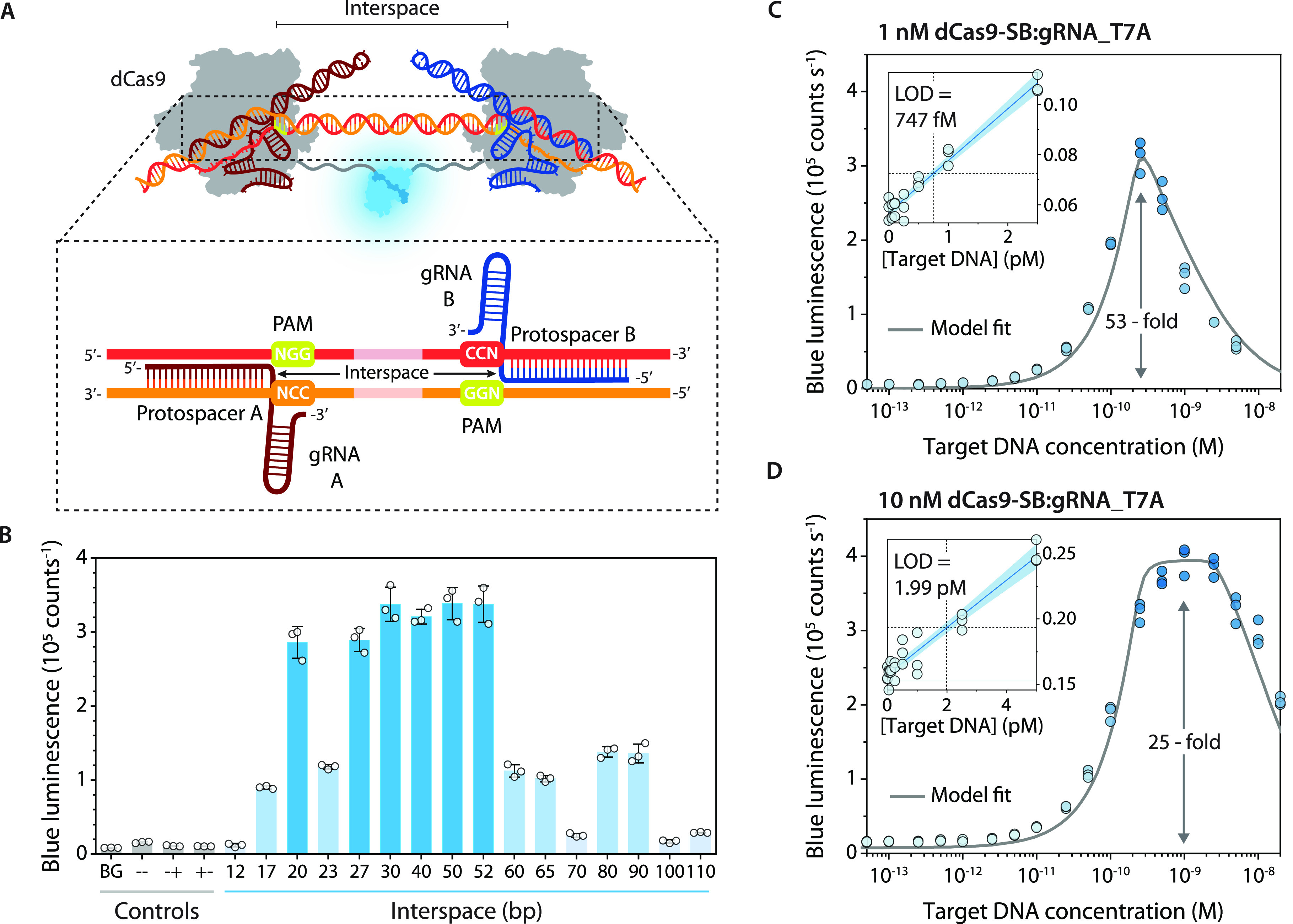
Characterization of LUNAS. (A) Schematic overview of the LUNAS complex. The zoomed-in view of the dsDNA hybridized with the two gRNAs shows the orientation of the gRNAs with respect to each other on the target DNA and indicates the interspace region between the PAM-proximal ends of the gRNAs. (B) Bar chart showing the blue luminescence (458 nm) observed with 1 nM of both dCas9-SB and dCas9-LB sensor RNPs for targets with various interspace lengths and negative controls containing DNA with no target protospacer (−−), only one (−+) or the other (+−), or no DNA at all (background, BG). (C, D) LUNAS response curve for a target with an interspace of 50 bp using (C) 1 nM dCas9-SB and dCas9-LB RNPs or (D) 10 nM dCas9-SB and 1 nM dCas9-LB RNP. A thermodynamic model was fitted to the data (gray solid line, see the Supporting Information). The limit of detection (LOD) was determined by local linear regression of sensor response at low target concentration (blue solid line with 95% confidence bands in inset). In B–D, individual data points (*n* = 3, technical replicates) are represented as circles, and bars in B represent means, with error bars showing SD.

Expression of dCas9-LB and dCas9-SB was performed in *E. coli*, and the proteins were purified by affinity column chromatography (Figure S3). To test the sensor and characterize its performance over a range of interspace distances between dCas9-SB and dCas9-LB, synthetic dsDNA fragments containing two protospacers on opposite strands spaced apart by 12–110 bp with PAMs facing inward were used as target (Figure 2A). We expected to attain the widest useful range of interspace distances using this protospacer orientation as the C-termini of the DNA-bound dCas9 proteins would face toward each other, minimizing the distance to be bridged by the peptide linkers to allow for SB – LB complementation.^34^ The dCas9-SB and dCas9-LB proteins were complexed with an excess of the complementary gRNAs (gRNA_T7A and gRNA_T7B, respectively) to form RNPs, and specific DNA binding was confirmed by an electrophoretic mobility shift assay (EMSA) (Figure S4). The RNPs (1 nM) were then incubated with target DNA (250 pM) for 30 min before the addition of furimazine substrate for signal detection. The sensor was found to yield maximal luminescent signal for interspace distances between 27 and 52 bp (Figure 2B). Shorter distances presumably lead to steric hindrance between the two RNPs, whereas longer interspaces cannot be effectively bridged by the linkers to allow for split NanoLuc complementation, both resulting in a low signal. Addition of control DNA lacking one or both of the protospacers did not result in any luciferase activation, confirming the sensor’s sequence specificity (Figure 2B). The remaining background luminescence is likely due to low levels of split NanoLuc complementation driven by the intrinsic affinity of SB for LB (Figure S5).

The concentration–response behavior of the sensor was examined by incubating 1 nM sensor RNPs with a range of target DNA concentrations (50 bp interspace) for 30 min, to allow for sufficient time to reach equilibrium (Figure S6). A robust ∼50-fold maximum increase over the background level was observed for 250 pM target DNA (Figure 2C). Higher DNA concentrations result in lower luminescence intensity, which is expected in the case of analyte excess as the chance of both sensor RNPs binding to the same DNA molecule decreases with increasing excess of DNA.^35^ Indeed, fitting a thermodynamic model of the system (see the Supporting Information) yields a bell-shaped curve in close agreement with the data (Figure 2C), assuming 25% of the RNPs is binding-competent, in accordance with previous reports on Sp(d)Cas9.^36,37^ Model simulations also show that this “hook” effect can be suppressed by increasing the relative amount of dCas9-SB RNP (Figure S2B), which we confirmed by measuring a concentration–response curve using 10 nM dCas9-SB and 1 nM dCas9-LB RNP (Figure 2D). The observed detection limit of 747 fM or 1.99 pM, using 1 or 10 nM dCas9-SB RNP respectively (Figure 2C, D), represents a substantial improvement in sensitivity over prior bioluminescent nucleic acid sensors with LODs of ∼6–50 pM.^26,27,31^ LUNAS is on par with fluorescent CRISPR Cas12- and Cas13-based methods excluding target preamplification that feature LODs of ∼1 pM to 5 nM depending on the specific Cas12/Cas13 orthologs used, with recently reported sensitivities down to 166 fM only achieved by measuring for 1–2 h.^11,38,39^

### Combining LUNAS with RPA

**Figure 3:**
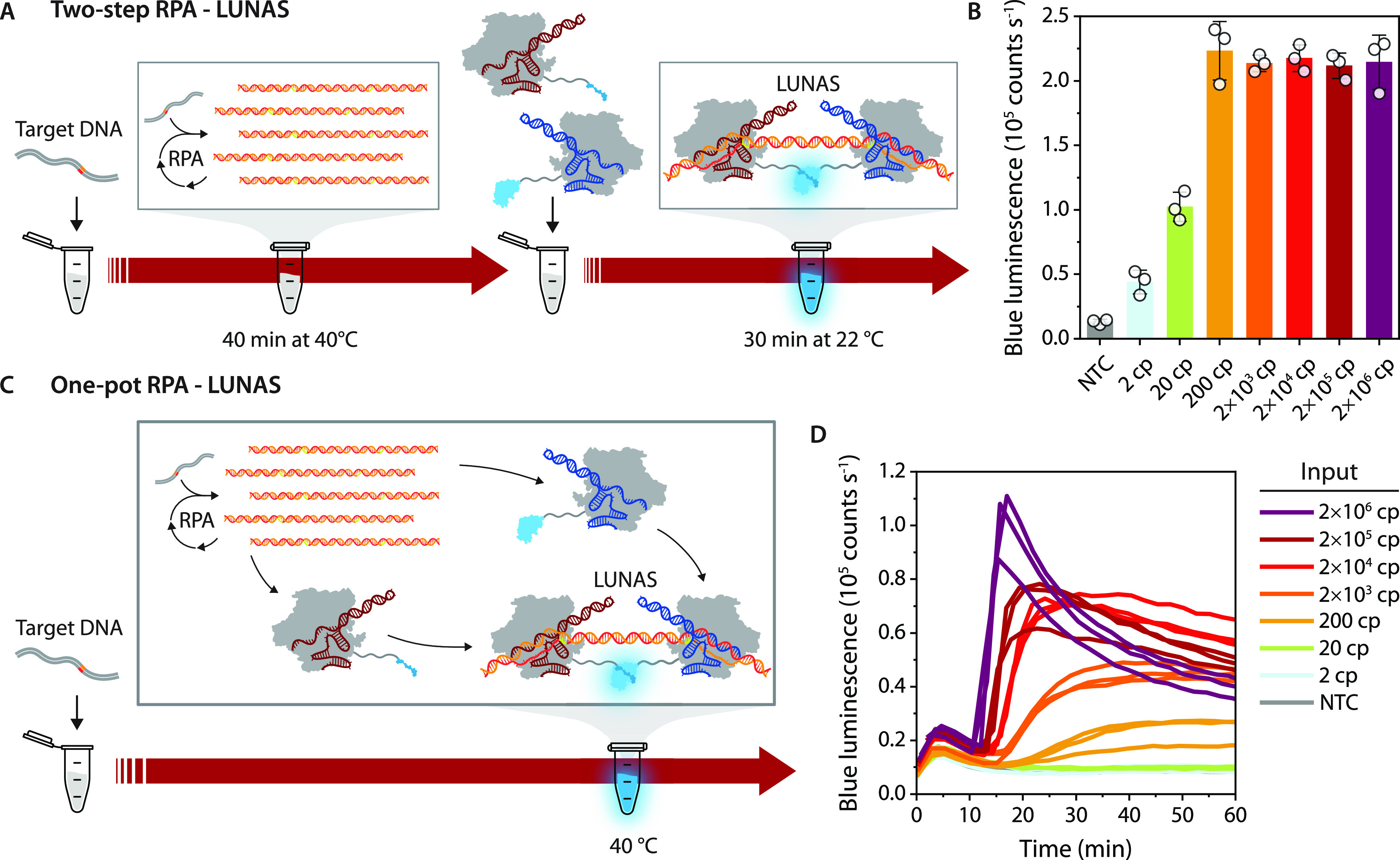
Combining LUNAS with RPA in two-step and one-step formats. (A) Schematic overview of the two-step RPA–LUNAS method in which the target DNA is first amplified in an RPA reaction, which is subsequently added to a LUNAS reaction mixture for end-point detection of the amplicons. (B) Results of the two-step RPA–LUNAS assay response for a range of synthetic target DNA inputs (1 μL), using 10 nM dCas9-SB:gRNA_T7A and 1 nM dCas9-LB:gRNA_T7B in a 20 μL reaction. Individual data points (*n* = 3, technical replicates) are represented as circles, and bars represent means, with error bars showing the SD. NTC: No template control. (C) Schematic overview of the one-pot RPA–LUNAS method in which both RPA and LUNAS components are present in the reaction mixture from the start, obviating postamplification reaction transfer and allowing monitoring of the RPA reaction in real-time. (D) Results of one-pot RPA–LUNAS assay response over time for the same range of target DNA inputs as used in B, using 10 nM dCas9-SB:gRNA_T7A and 1 nM dCas9-LB:gRNA_T7B. Individual replicate traces (*n* = 3) are shown.

Although the ∼1 pM limit of detection of LUNAS is excellent for a direct nucleic acid sensor, the detection of pathogen nucleic acids from diagnostic samples typically requires sensitivity in the attomolar concentration range.^28−30^ The sensitivity of LUNAS cannot be improved much further, as it is limited by the minimal amount of reconstituted luciferase required for producing sufficient signal, which is on the order of 100 fM (Figure S7). Therefore, we explored combining LUNAS with isothermal amplification of the target nucleic acid. We selected RPA for its rapid exponential amplification at a relatively low optimal temperature of 37–42 °C, a temperature at which both dCas9 and split NanoLuc are stable.^32,40,41^ RPA primers were designed for one of the synthetic LUNAS target DNA fragments used in Figure 2 (30 bp interspace). Initially, a two-step protocol was tested by first amplifying minute concentrations of target DNA during a 40 min RPA reaction, followed by incubation of this reaction with the luminescent sensor mixture for 30 min at room temperature and subsequent measurement of luminescence intensity upon addition of substrate (Figure 3A). This method provided excellent sensitivity, showing an increase in blue luminescence for inputs down to 2 copies of target DNA, detecting 3.3 aM in a 1 μL sample (Figure 3B). The assay response reached a plateau at an input ≥200 copies, which suggests that the RPA reaction was exhausted, yielding the same maximal amplicon output for higher input copy numbers.^42^

Next we explored whether RPA and LUNAS amplicon detection could be performed simultaneously in a one-pot reaction. For this, the LUNAS reaction mixture including substrate was added to the RPA reaction with RPA components at default concentrations, and luminescence was monitored while incubating the 20 μL mixture at 40 °C (Figure 3C). Considering the multiple molecular interactions involved, with potential competition between binding of the dCas9 RNPs and components of the RPA reaction (recombinases, DNA polymerase, ssDNA binding proteins), the assay performed remarkably well. After an initial “bump” in luminescent signal, likely induced by the temperature increase in the wells, a sharp rise in signal was observed for high target input concentrations within 20 min, whereas target inputs down to 200 copies could still be clearly distinguished from the nontarget control within 30 min (Figure 3D).

### RT-RPA-LUNAS for SARS-CoV-2 RNA Detection

**Figure 4:**
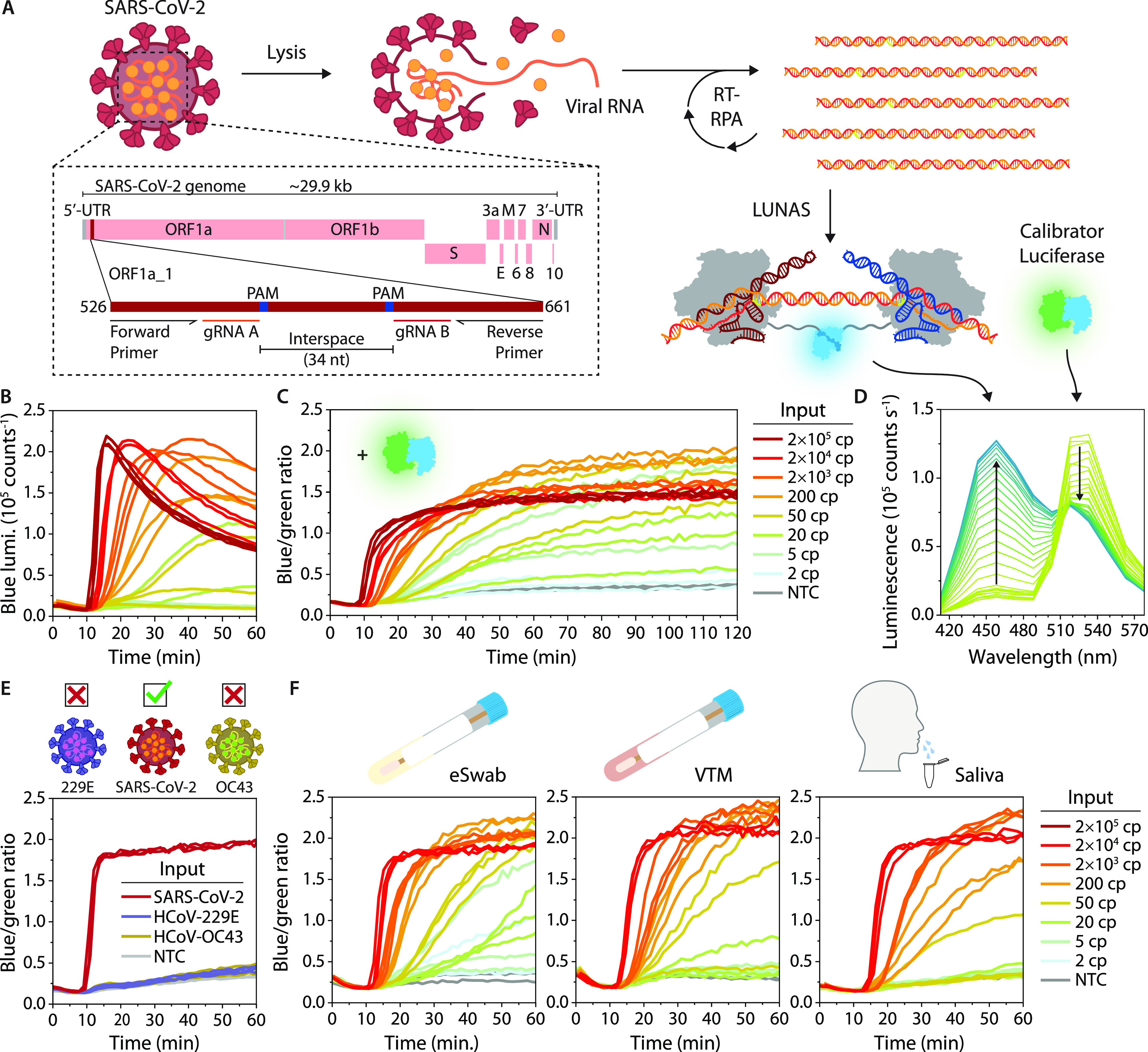
SARS-CoV-2 RT-RPA-LUNAS assay. (A) Schematic representation of the SARS-CoV-2 RT-RPA-LUNAS assay, showing the viral genome and zooming in on the assay target region (ORF1a_1) within ORF1a (see Table S1 for primer and gRNA sequences). (B) Intensiometric one-pot RT-RPA-LUNAS response over time for a range of IVT ORF1a RNA inputs (see legend in C). (C) Ratiometric one-pot RT-RPA-LUNAS response over time for a range of IVT ORF1a RNA inputs. (D) Luminescence spectra of a single ratiometric one-pot RT-RPA-LUNAS reaction from panel C (200 cp input), showing one line per time point over the first 45 min, with trends indicated by arrows (see also Figure S11). (E) Ratiometric one-pot RT-RPA-LUNAS response over time for genomic RNA (2 × 10^4^ cp) isolated from SARS-CoV-2 (target) and from closely related HCoV-229E and HCoV-OC43 nontarget viruses, showing the specificity for the target. (F) One-pot RT-RPA-LUNAS compatibility tests for eSwab, VTM and saliva sample matrices, showing the ratiometric RT-RPA-LUNAS response over time for the same range of IVT ORF1a inputs as in D. Mixtures of the sample media and inactivation buffer (1:1) were spiked with IVT target RNA and heated at 95 °C for 5 min (eSwab, saliva) or 70 °C for 10 min (VTM) (see methods) before addition to the RT-RPA-LUNAS reactions. In B, C, E, and F, individual replicate traces (*n* = 3) are shown. NTC: No template control.

Having achieved attomolar sensitivity in a one-pot method, we set out to design an RPA-LUNAS assay for the detection of a clinically relevant target. Triggered by the COVID-19 pandemic, we designed gRNAs and RPA primers for the SARS-CoV-2 RNA genome (Wuhan-Hu-1), taking into account prevalent nucleotide mutations known at the time and limiting homology with genomes of related human coronaviruses including common cold viruses HCoV-OC43, HCoV-229E, HCoV-HKU1, and HCoV-NL63. Designed gRNA and primer sets targeting three different genomic regions were screened for the best performance using corresponding cDNA target fragments (Figure S8). The resulting best performing assay targets open reading frame 1a (ORF1a), with primers flanking the sensor RNP binding sites (Figure 4A). To allow for RNA detection, a reverse transcriptase was next included in the reaction mixture as well as RNase H, which degrades the RNA in DNA:RNA hybrids and was previously shown to enhance the sensitivity of RT-RPA reactions.^14,43^ The resulting one-pot LUNAS assay was found to reliably detect as little as 200 copies of in vitro transcribed (IVT) ORF1a RNA fragment input in a 20 μL reaction within 25 min (Figure 4B), with the two-step equivalent providing a further ∼10-fold increase in sensitivity (Figure S9). Lower inputs showed a stochastic assay response, with only some replicates yielding a detectable increase in signal.

In the one-pot assay, the luminescence intensity gradually decreases over time after reaching a peak level, especially for higher intensity signals. This time-dependence is nonideal, in particular for point-of-care applications. First we considered the possibility that the decrease in signal was a reflection of the “hook” effect observed in the titration experiment in Figure 2C, with amplicon accumulation leading to a decrease in signal upon redistribution of sensor RNPs over the larger number of amplicons. However, once bound to its target DNA, Sp(d)Cas9 is known to dissociate only very slowly, effectively staying tightly bound for hours.^44,45^ We therefore performed an experiment in which the LUNAS sensor components were first incubated with an optimal concentration of target DNA (250 pM) for 1 h. Subsequently, we increased the DNA target concentration further to 2.5 nM and monitored the luminescence intensity. The rate of decrease in luminescence was not substantially faster compared to a control left at the initial target DNA concentration (Figure S10), indicating that binding of Sp(d)Cas9 RNP is indeed kinetically controlled. The decrease in luminescence intensity in RPA-LUNAS assays is therefore unlikely to be the result of redistribution of sensor RNPs over the larger number of amplicons. The decrease in absolute intensity over time may also be explained by gradual substrate depletion. Our group recently described the use of a green light emitting mNeonGreen-NanoLuc (mNG-NL) fusion protein, in which NanoLuc acts as a BRET donor to excite mNeonGreen, as a means for direct internal calibration of the RAPPID bioluminescent immunoassay.^25,46^ Since the activity of this calibrator luciferase is equally dependent on the substrate concentration, including this calibrator luciferase in the RPA-LUNAS assay and taking the blue-over-green emission ratio yields an output corrected for substrate depletion. Indeed, doing so resulted in a ratiometric response that was stable in time, ruling out alternative explanations^47,48^ and simplifying result interpretation (Figure 4C, D). Using this ratiometric assay, inputs as low as 5 cp could be detected, although stochasticity was observed for inputs <50 cp (Figure 4C).

To verify the specificity of the RT-RPA-LUNAS assay, the responses for full genomic RNA isolates from SARS-CoV-2 as well as related HCoV-OC43 and HCoV-229E were compared in a one-pot experiment. Evidently, no increase in emission ratio was observed for all but the target input (Figure 4E). With the aim of validating the assay using COVID-19 patient samples, we next explored suitable sample preparation methods. The typical RT-qPCR workflow involves chemical lysis and magnetic bead or filter based RNA isolation, which allows for control over the elution buffer and concentration of the RNA. However, in a POC setting, a test should ideally require only a simple viral inactivation step to make the viral RNA accessible for detection. Successful heat-inactivation methods have previously been described for SARS-CoV-2 RNA detection from VTM (Viral Transport Medium), used for nasopharyngeal swab collection, and saliva.^14,43^ In these methods, an inactivation buffer containing TCEP and an RNase inhibitor is used to effectively inactivate omnipresent RNases, which are notorious for being especially stable. To explore extraction-free detection from typical COVID-19 diagnostic sample matrices, we tested the compatibility of our assay with VTM, liquid Amies (eSwab), and saliva inputs. An inactivation buffer (100 mM TCEP, 1 mM EDTA, 1U/μL murine RNase inhibitor, 10 mM Tris-HCl, pH 8.0) was added to the sample medium, and IVT target RNA was added before heating the mixture to 95 °C for 5 min (eSwab, saliva) or 70 °C for 10 min (VTM). As a control, additional saliva samples were prepared without the addition of the inactivation buffer. For subsequent detection, 1 μL of a mock sample was used as input for a 20 μL RT-RPA-LUNAS reaction. In this way, target RNA could be detected from all three sample types, with the highest sensitivity for the eSwab sample, and approximately 10-fold lower sensitivity for saliva input (Figure 4F). RNase inactivation is crucial in this procedure as no target RNA was detected in saliva samples treated with heat only (Figure S12).

### SARS-CoV-2 Assay Validation

**Figure 5:**
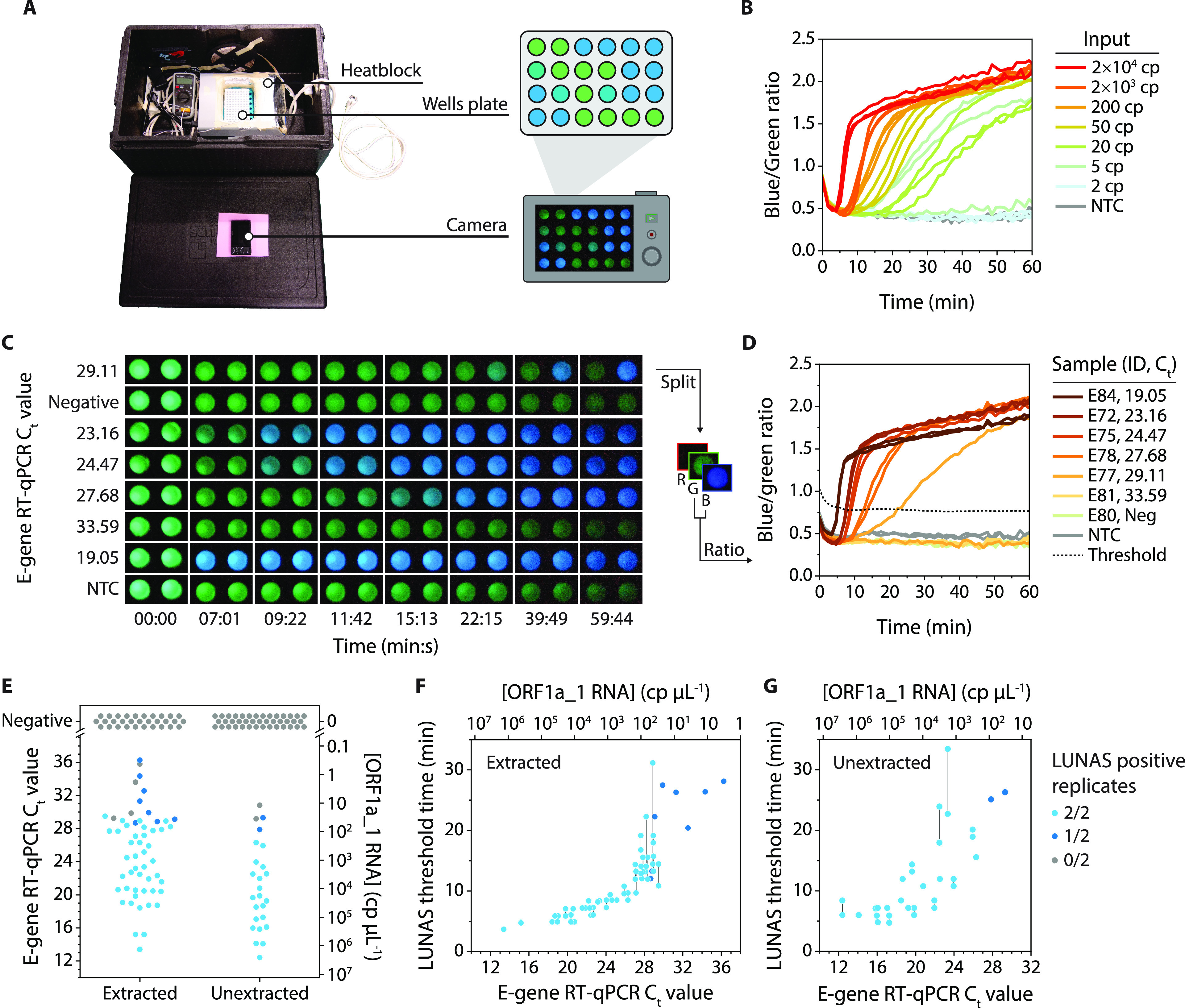
Detection of SARS-CoV-2 from patient samples. (A) Experimental setup as used for camera-based readout of the ratiometric RT-RPA-LUNAS assay, consisting of a dark box with a simple heat block to keep a white 96-well plate at 40 °C, and a digital camera fitted through a hole in the lid to continuously record pictures of the reactions. (B) Camera-based readout of experiment that is similar to the one shown in Figure 4F (eSwab), with the ratiometric RT-RPA-LUNAS response extracted from pictures recorded by the camera. Individual replicate traces (*n* = 3) are shown. (C) Pictures of a subset of ratiometric RT-RPA-LUNAS reactions with clinical sample inputs (RNA isolates), showing the blue/green intensity at several points in time. Two wells shown within the same dark box represent technical duplicates. (D) Blue/green ratio traces over time as extracted from the RGB images in (C). Individual traces are shown for both duplicates. The dashed line represents the threshold blue/green ratio and is based on the NTC and variance among all reactions (see methods). Also see timelapse in Movie S1. NTC = No template control (E–G) Summary of RT-RPA-LUNAS test results from two independent groups of clinical samples: RNA extracted from nasopharyngeal swabs (“extracted”) and eSwab samples that were treated according to our heat-inactivation protocol (i.e., “unextracted”). Colors code for the RT-RPA-LUNAS outcome, and known E-gene RT-qPCR *C*_t_ values as well as the absolute concentration of the target region (ORF1a_1) in the original sample material predicted based on RT-ddPCR data for a subset of samples (Figure S14, Table S2) are shown on the axes. (E) Overview of RT-RPA-LUNAS outcomes for all tested samples, showing a single data point per sample. (F) RT-RPA-LUNAS threshold time for all tested RNA isolates extracted from COVID-19 patient samples (see methods). (G) RT-RPA-LUNAS threshold time for all tested unextracted eSwab COVID-19 patient samples (see methods). In F and G, data points represent individual replicates, with a vertical solid line connecting nonoverlapping duplicates.

To clinically validate RT-RPA-LUNAS, we tested the diagnostic performance for nasopharyngeal patient samples. We used an experimental setup using a heating block and a standard digital camera for signal detection, illustrating simple execution of the assay with minimal equipment (Figure 5A). The camera-based readout was tested using the same conditions as for Figure 4F, and similar responses were observed with emission ratios calculated from blue- and green-channel intensities in RGB pictures (Figure 5B and Figure S13). With this setup, an increase of blue signal was observed already after just 5 min for the higher concentrations, most likely reflecting the more efficient conductive heat transfer when using the heat block compared to the convective heating in the plate reader.

Using the camera-based readout, we next tested RT-RPA-LUNAS on clinical nasopharyngeal samples. Both RNA isolates (*N* = 86, of which 55 were positive), as well as eSwabs (*N* = 66, of which 25 were positive) treated by our heat-inactivation protocol directly prior to the assay, were included, constituting two independent sample groups for which comparator RT-qPCR results were known. To normalize and convert these *C*_t_ values to concentrations of the target RNA fragment, droplet digital PCR (RT-ddPCR) was performed for a selection of samples (Figure S14). Using the RT-RPA-LUNAS assay, the samples were measured in duplicate, adding only 1 μL per 20 μL reaction, and were considered positive for SARS-CoV-2 if the blue/green emission ratio surpassed the nontarget control level by a threshold margin based on the variance among all reactions at *t* = 1–3 min (Figure 5C, D). Specifically, the threshold blue/green ratio (BGR_T_) is defined as BGR_T_(*t*_*i*_) = BGR_smoothNTC_(*t*_*i*_) + 6SD_All,1–3 min_ for *t*_*i*_ to *t*_*i*+2_, with BGR_smoothNTC_(*t*_*i*_) the moving average blue/green ratio of the NTC over (*t*_*i*–5_, *t*_*i*+5_), and SD_All,1–3 min_ the standard deviation of the blue/green ratio of all reactions between *t* = 1 min and 3 min. The reported LUNAS threshold time t_T_ equals the first *t*_*i*_ satisfying the condition BGR(*t*_*i*_) > BGR_T_(*t*_*i*_) for (*t*_*i*_, *t*_*i*+2_). All tested RNA isolates with an E-gene RT-qPCR *C*_t_ value <28.5 (>56 cp/μL; > 86 cp input) were identified as positive for SARS-CoV-2 by LUNAS within 22 min, with those having *C*_t_ < 27 (>154 cp/μL; >231 cp input) detected within 11 min (Figure 5E, F and Figure S15). The assay also performed well for the nonextracted samples, although based on the limited number of samples tested in the higher *C*_t_ value range the sensitivity appears to be slightly lower, correctly identifying all tested RT-qPCR-positive samples up to *C*_t_ ≈ 26.5 (>215 cp/μL; >107 cp input) (Figure 5E, G, and Figure S16). We presume this difference in apparent limit of detection to be largely attributable to the 1.5-fold increase in RNA concentration occurring as part of the isolation procedure for the extracted samples versus the 2-fold dilution of samples in our heat inactivation protocol (see methods). For both types of input, all true negatives tested were indeed identified as such, including those positive for other respiratory viruses (Table S2). In samples for which the comparator assay reported a high *C*_t_ value, either one or both of the replicate reactions failed to signal the presence of SARS-CoV-2 RNA, correctly identifying 21 out of 25 positive eSwabs and 43 out of 55 positive RNA isolates in both replicates in total. Such stochasticity can also be observed for similar inputs (≤50 cp) of IVT target RNA (Figure 4B, C, F), indicating similar sensitivity of the assay for the mock and the clinical samples. These results show that RT-RPA-LUNAS approaches the sensitivity of RT-qPCR, while providing important benefits with respect to assay time and required instrumentation, demonstrating its potential for point of care applications.

## Discussion

The LUNAS platform reported here provides a highly sensitive and generally applicable bioluminescent platform for sequence-specific dsDNA detection that is ideally suited for use in combination with isothermal amplification methods such as RPA. RPA-LUNAS rivals other recently developed isothermal NA amplification- and CRISPR-based diagnostic methods in terms of speed, specificity, and sensitivity, while providing in-sample bioluminescent detection that is particularly attractive for use in low resource settings. We showed that dCas9-split-NanoLuc-based nucleic acid detection can be combined with RPA in an efficient one-pot assay with real-time readout, avoiding the risk of cross-contamination due to postamplification reaction transfer. This is in contrast to lateral flow assay readouts or two-step fluorescent approaches commonly used in isothermal amplification and CRISPR-based NA detection methods, which require transfer of the amplification reaction to the lateral flow device or the CRISPR cleavage reaction.^14,15,43^ The readout of RPA-LUNAS is further simplified by including the previously developed mNeonGreen-NanoLuc calibrator luciferase^25,46^ to generate a robust ratiometric output, enabling straightforward signal recording by a digital camera.

With two guide RNAs and two RPA primers that govern target recognition, covering a total of ∼100 nucleotides, RPA-LUNAS is highly specific while also being easily programmable. Provided a 5′-*NGG-*3′ PAM and its reverse complement 5′-*CCN*-3′ exist within 27–52 nt of each other in a nucleic acid strand, this method can be readily adapted to detect such a target by designing corresponding gRNAs and RPA primers. As proof of principle, we demonstrated the use of RPA-LUNAS for the detection of SARS-CoV-2 RNA from nasopharyngeal samples of COVID-19 patients. Employing a simple heat-inactivation protocol, we showed that LUNAS is able to reliably identify COVID-19 positive cases without RNA isolation for samples with a viral load >200 cp/μL, mostly within 20 min. This is on par with other CRISPR diagnostic methods applied to SARS-CoV-2 detection, such as the recently improved one-pot fluorescent SHERLOCK method (SHINE), which achieved similar sensitivity in 40 min (see Table S3).^14^ For the specific case of the SARS-CoV-2 assay, the sensitivity can likely be improved further by increasing the sample input volume, decreasing the volume of the swab collection medium, or by higher-fold concentration in an RNA extraction step. Although progress has been made in simplifying RNA isolation in recent years, current methods are still cumbersome, leaving room for improvement for application in POC settings.^49^

The one-pot (RT-)RPA-LUNAS assay is remarkably effective considering the multiple potential interferences between reaction components, featuring a detection limit that is only ∼10-fold higher than that of the two-step assay. This small difference in intrinsic sensitivity may be further reduced by an additional optimization of the reaction conditions. The presence of dCas9 RNPs from the start of the amplification reaction could potentially block RPA for low target concentrations due to tight binding of the dCas9 complexes to the scarce templates. Such an effect has been observed for RPA/Cas12-based NA detection, and was recently resolved by introducing a photoactivatable guide RNA that allows for delayed activation of RNPs.^50,51^ Similarly, targeting suboptimal PAMs to slow down Cas12 cleavage kinetics was recently shown to benefit overall reaction efficiency in an RPA/Cas12a-based assay.^52^

To enable multiplexing in a single reaction, a green or red color variant of the LUNAS system could be developed by the use of BRET, analogous to the mNG-NL calibrator luciferase. Such a color variant could be used as part of a build-in control mechanism for appropriate sample collection to detect an endogenous NA that is amplified alongside the target NA in a duplex RPA reaction.^18,53^

The simple assay setup, with only a brief heat inactivation step for in-sample viral NA detection, is expected to allow for relatively straightforward integration of one-pot (RT-)RPA-LUNAS in a cheap and portable diagnostic device, that can be readout using a smartphone camera. Making use of advances in microfluidics and miniature electronic or chemical heating devices, integrated sample preprocessing, and lyophilization of the reaction mixture could further streamline the assay.^18,54,55^ In conclusion, RPA-LUNAS shows great potential for translation into a sensitive and rapid point-of-care nucleic acid diagnostic that can readily be reconfigured for a quick diagnostic response following an epidemic outbreak.

## Data Availability

All data underlying the presented figures and findings is available at 10.4121/21581547. The gRNA design tool that functions as an add-on to the CRISPOR tool developed by Haeussler et al.^56,57^ is available in a GitHub repository (https://github.com/harmveer/LUNAS_CRISPOR_tool). Plasmids for recombinant expression of dCas9-SB (Addgene #198750) and dCas9-LB (Addgene #198751) will be made available through Addgene.

## Supporting Information Available

Materials and Methods, ethics and safety statement, protein coding sequences, and supplementary figures and tables (PDF)
