# Supplementary material for: Glow-in-the-Dark Infectious Disease Diagnostics Using CRISPR-Cas9-Based Split Luciferase Complementation
Source: ACS Cent Sci. 2023 Mar 15;9(4):657–67. doi: 10.1021/acscentsci.2c01467 (PMC10141630; doi:10.1021/acscentsci.2c01467)
Supplement: Supplementary file 1 — oc2c01467_si_001.pdf [file oc2c01467_si_001.pdf]

Glow-in-the-dark infectious disease diagnostics using CRISPR-Cas9-based split luciferase complementation

Harmen J. van der Veer[1,2], Eva A. van Aalen[1,2], Claire M. S. Michielsen[1,2], Eva T. L. Hanckmann[1,2], Jeroen Deckers[1,2], Marcel M. G. J. van Borren[3], Jacky Flipse[4], Anne J. M. Loonen[5,6], Joost P. H. Schoeber[5], & Maarten Merkx[1,2,*].

[1] Laboratory of Chemical Biology, Department of Biomedical Engineering, Eindhoven University of Technology, P.O. Box 513, 5600 MB, Eindhoven, The Netherlands.
[2] Institute for Complex Molecular Systems, Eindhoven University of Technology, P.O. Box 513, 5600 MB, Eindhoven, The Netherlands.
[3] Department of Clinical Chemistry, Rijnstate Hospital, P.O. Box 9555, 6800 TA, Arnhem, The Netherlands.
[4] Laboratory for Medical Microbiology and Immunology, Rijnstate Hospital, P.O. Box 8, 6880 AA, Velp, The Netherlands.
[5] Research Group Applied Natural Sciences, Fontys University of Applied Sciences, 5612 AP, Eindhoven, The Netherlands.
[6] Pathologie-DNA, Lab for Molecular Diagnostics, Location Jeroen Bosch Hospital, 5223 GZ, 's-Hertogenbosch, The Netherlands.
* To whom correspondence should be addressed. Email: m.merkx@tue.nl

Supporting Information

This PDF file includes:

Material and Methods
Figures S1 to S16
Table S3
Caption for Movie S1
Supporting Information References

Other supporting data for this manuscript include the following:

Tables S1 and S2
Movie S1

Contents

Material and Methods....S3
Cloning and protein expression....S3
Assay design and synthetic target nucleic acids....S3
Electrophoretic mobility shift assay (EMSA)....S4
LUNAS assays....S4
(RT-)RPA-LUNAS assays....S5
Clinical validation....S5
RT-qPCR and ddPCR testing of clinical samples....S7
Thermodynamic model....S7
Figure S1: Model of the thermodynamic interactions in the LUNAS assay....S8
Figure S2: Model simulations of LUNAS response for a variation in sensor parameters....S10
Ethics statement....S10
Safety statement....S10
Figures....S11
Figure S3: Expression and purification of LUNAS proteins....S11
Figure S4: Electrophoretic mobility shift assay (EMSA) with LUNAS RNPs....S12
Figure S5: LUNAS background luminescence....S13
Figure S6: Kinetics of intensiometric LUNAS....S14
Figure S7: NanoLuc titration curve....S15
Figure S8: RPA-LUNAS SARS-CoV-2 assay gRNA and primer screening....S16
Figure S9: 2-step RT-RPA-LUNAS SARS-CoV-2 assay....S17
Figure S10: LUNAS kinetics upon increase in target concentration following initial equilibration...S18
Figure S11: Ratiometric RPA-LUNAS luminescence spectra over time....S19
Figure S12: Importance of RNase inactivation for RT-RPA-LUNAS in saliva samples....S20
Figure S13: Camera-based RT-RPA-LUNAS for SARS-CoV-2 RNA detection from saliva....S21
Figure S14: RT-ddPCR quantification of SARS-CoV-2 RNA in clinical samples....S22
Figure S15: RT-RPA-LUNAS ratiometric response traces of all extracted clinical samples....S23
Figure S16: RT-RPA-LUNAS ratiometric response traces of all unextracted clinical samples....S24
Tables....S25
Table S3: Comparison of RT-RPA-LUNAS SARS-CoV-2 assay performance with that of other recent CRISPR diagnostic methods applied to SARS-CoV-2 detection....S25
Movie....S26
Movie S1 (separate file). Timelapse of an RT-RPA-LUNAS assay detecting SARS-CoV-2 RNA in clinical samples....S26
Protein coding sequences and translations....S27
dCas9-SB / dCas9-LB....S27
mNG-NL calibrator luciferase....S30
References....S31

Material and Methods

Cloning and protein expression

The *S. pyogenes* dCas9 coding sequence was copied from the pET-dCas9-VP64-6xHis plasmid gifted by David Liu (Addgene plasmid #62935) by means of overhang PCR and was cloned via traditional restriction/ligation into a pET28a(+) plasmid (ordered from GenScript) coding for the C-terminal flexible linker and small BiT (SB) as well as large BiT (LB) and a C-terminal Strep-tag II (see protein coding sequence in Supporting Information). *E.coli* BL21 (DE3) was transformed with the resulting plasmid directly for dCas9-SB expression, whereas the [SB – Strep-tag II – stop codon] sequence in-between the flexible linker and LB coding portions was removed by restriction/ligation for dCas9-LB expression. All cloning was confirmed successful by Sanger sequencing (BaseClear). Both proteins were expressed in *E.coli* BL21 (DE3), grown in LB-Miller medium with kanamycin (50 µg/mL) to OD600 = 0.6 – 0.8 at 37°C before induction by 0.2 M IPTG. Following overnight incubation at 18°C, cells were harvested by centrifugation (8600×g, 15 min., 4°C) and resuspended in 12.5 mL pre-chilled lysis buffer (500 mM NaCl, 1 mM TCEP, 50 mM Tris-Cl pH 8.0) per gram of cell pellet, supplemented with benzonase (25 U/mL) (Merck) and a cOmplete EDTA-free protease inhibitor cocktail tablet (Merck). Cells were lysed by 3 passes through a high pressure homogenizer (Avestin Emulsiflex C3), at 15’000 – 20’000 Psi. The proteins were purified using Strep-Tactin XT (Iba) purification. Protein purity was confirmed by reducing SDS-PAGE (see SI) and concentrations were determined by measurement of absorbance at 280 nm on a NanoDrop spectrometer using extinction coefficients calculated from the protein sequence. Proteins in Strep-Tactin XT elution buffer (150 mM NaCl, 100 mM Tris-Cl pH 8.0, 1 mM EDTA, 50 mM D-biotin, 1 mM TCEP) were aliquoted and snap frozen in liquid nitrogen and stored at −70°C.

The mNG-NL calibrator luciferase was expressed and purified as described previously [1], and the same procedure was followed for expression and purification of NanoLuc from a plasmid available in our lab (for Figure S7).

Assay design and synthetic target nucleic acids

Guide RNA pairs were designed using the CRISPOR tool developed by Haeussler et al. [2,3] and a custom add-on tool (LUNAS_CRISPOR_tool) taking into account the on-target activity as predicted by the scoring algorithms described by Moreno-Mateos et al. [4] and Doench et al. [5], as well as the specificity scores based on Hsu et al. [6] and Doench et al. [5]. For the initial LUNAS assay used for sensor characterization, we designed 2 gRNAs that target bacteriophage T7 protospacers. These target sites were included in synthetic target DNA fragments with varying interspace distance separating the two protospacers. gRNA was generated from crRNA + tracrRNA (IDT) by combining both 1:1 in IDT nuclease free duplex buffer (30 mM HEPES, pH 7.5; 100 mM potassium acetate) to 4 µM final gRNA duplex concentration and heating at 95°C for 5 min., followed by gradually cooling to room temperature. gRNAs were aliquoted and stored at −30°C. Target DNA fragments were ordered as gBlocks (IDT) or PCR amplified from plasmids containing multiple such fragments, and then gel purified. For initial RPA-LUNAS experiments (Figure 3), RPA primers were designed for these synthetic targets, following TwistAmp (TwistDx) primer design guidelines.

For the SARS-CoV-2 assay, the genome of the original Wuhan-Hu-1 isolate (GenBank: NC_045512.2) was scanned for suitable target site pairs using the CRISPOR tool [2,3] and our LUNAS add-on. Candidate gRNAs were screened for specificity against genomes of related common cold human coronaviruses OC43, NL63, HKU1 and 229E as well as SARS-CoV and MERS-CoV (NCBI RefSeq accessions NC_006213.1; NC_005831.2; NC_006577.2; NC_002645.1; NC_019843.3; NC_004718.3 respectively). Three gRNA pairs predicted to be highly specific and having individual Moreno-Mateos [4] activity scores >30/100 were selected. Complementary RPA primers were designed using the PrimedRPA tool developed by Higgins et al. [7], aiming for small amplicon size. Final assay designs were checked for the absence of SNPs with >1% prevalence known at the time based on GISAID [8]/Nextstrain [9] data in the UCSC SARS-CoV-2 genome browser [10].

Corresponding SARS-CoV-2 cDNA fragments, PCR amplified from positive control plasmids provided by the FreeGenes project, were used for gRNA screening in LUNAS assays and subsequent

primer screening in RPA-LUNAS assays. Synthetic ORF1a target RNA fragment was produced from the corresponding cDNA fragment by in vitro transcription using the HiScribe T7 High Yield RNA Synthesis Kit (NEB), according to manufacturer's instructions. The IVT reaction was treated with DNAse I (Thermo Fisher) to degrade template DNA according to the manufacturer's instructions. The IVT RNA was purified using a Monarch RNA cleanup spin column kit (NEB) and aliquoted for storage at -70°C. Concentrations of all nucleic acids were determined based on NanoDrop absorbance measurement at 260 nm (with 1 A260 optical density unit equal to 40 µg/mL RNA or 50 µg/mL dsDNA).

In addition, SARS-CoV-2 (Isolate USA-WA1/2020, BEI catalog No. NR-52347), HCoV-OC43 (BEI catalog No. NR-52727) and HCoV-229E (BEI catalog No. NR-52728) genomic RNA extracted from heat-inactivated virus was obtained from BEI Resources at known concentrations as determined by ddPCR.

The sequences of all crRNAs, RPA primers and synthetic targets are listed in Table S1. The gRNA design tool that functions as an add-on to the CRISPOR tool [2,3] is available at https://github.com/harmveer/LUNAS_CRISPOR_tool.

Electrophoretic mobility shift assay (EMSA)

dCas9-SB:T7A and dCas9-LB:T7B RNP complexes were preassembled by incubating dCas9-SB with T7A gRNA and dCas9-LB with T7B gRNA separately in LUNAS buffer (20 mM Tris-Cl pH 7.5, 150 mM KCl, 5 mM $MgCl_2$, 5% (v/v) glycerol, 1 mM DTT, 1 mg/mL BSA) for 15 min at 37°C, combining 1 µM protein with a 3-fold excess of gRNA. The EMSA was performed by incubating 12.5 nM target (473 bp) or non-target DNA (510 bp) fragment with 0.5 – 16 molar equivalents of dCas9-SB:T7A and/or dCas9-LB:T7B complexes in LUNAS buffer for 1 hour at room temperature. For subsequent electrophoresis, the reactions were supplemented with 1x loading dye (no SDS, NEB) and loaded onto a 2% agarose gel including 1x SYBR Safe, along with a 100 bp GeneRuler ladder (Thermo Fisher). Electrophoresis was performed for 1 h at 100 V in TAE running buffer (40 mM Tris, 20 mM acetic acid, 1 mM EDTA, pH 8.4). Bands were visualized on a Cytiva ImageQuant 800 using default settings for fluorescence imaging with automatic exposure adjustment.

LUNAS assays

For LUNAS experiments, dCas9-SB:gRNA_A and dCas9-LB:gRNA_B complexes were preassembled separately using fresh protein and gRNA stock aliquots on the day of use by incubating dCas9-SB/LB (10 – 100x final sensor concentration) with the corresponding gRNA (3-fold excess) in LUNAS buffer (20 mM Tris-Cl pH 7.5, 150 mM KCl, 5 mM $MgCl_2$, 5% (v/v) glycerol, 1 mM DTT, 1 mg/mL BSA) for 15 min at 37°C. LUNAS assays (without RPA) were performed at sensor RNP complex concentrations of 1 – 10 nM in a total volume of 20 µL, in Nunc 384-well non-treated flat-bottom white microplate (Thermo Fisher). Input DNA was prepared by serial dilution in LUNAS buffer, of which 1 µL was added to a LUNAS reaction. For the interspace variation and DNA titration assays (Figure 2), 1 µL NanoGlo substrate (Promega, N1110) was added at a final dilution of 2100-fold after 30 min incubation at room temperature. Luminescence spectra (398 nm – 653 nm, step size 15 nm, bandwidth 25 nm) were recorded on a Tecan Spark 10M plate reader with an integration time of 100 – 200 ms and data was collected using Tecan SparkControl V2.1 / V3.1. 'Blue' luminescence refers to the luminescence intensity at 458 ± 12.5 nm. LODs were calculated in Microsoft Excel by linear regression of sensor response over a limited concentration range, using the standard deviation of the y-intercept [11].

For the kinetic measurements (Figure S6, S10), NanoGlo (1000-fold final dilution) was directly included upon mixing sensor RNP complexes and input DNA in a total reaction volume of 20 µL, and luminescence spectra were recorded over time.

(RT-)RPA-LUNAS assays

For the 2-step (RT-)RPA-LUNAS assays, RPA reactions were prepared on ice using the TwistAmp Basic kit (TwistDx), first making a master mix comprising 505.26 nM of both primers, 14.74 nM magnesium acetate and 62.11% (v/v) TwistAmp rehydration buffer. For RT-RPA reactions, the master mix

additionally included 2.11 U/µL SuperScript IV reverse transcriptase (Invitrogen), 0.105 U/µL RNase H (NEB) and 1.05 U/µL murine RNase inhibitor (NEB). This master mix was used to resuspend lyophilized RPA reaction components (TwistAmp Basic kit, TwistDx) at 47.5 µL per pellet. RPA reactions were performed at 40°C for 40 min in a total volume of 20 µL, combining 1 µL sample with 19 µL reaction mixture per replicate in a 96-well white PCR plate (VWR). For amplicon detection, a 1 µL sample was added to LUNAS reactions prepared and performed as described above.

For one-pot RPA-LUNAS assays, reactions were prepared on ice by first making a master mix containing 505.26 nM primers, 1053-fold diluted NanoGlo substrate, 14.74 nM magnesium acetate, 62.11% (v/v) TwistAmp rehydration buffer (TwistAmp Basic kit, TwistDx), and 10.53% (v/v) LUNAS mix (100 nM dCas9-SB:gRNA_A and 10 nM dCas9-LB:gRNA_B in LUNAS buffer). For ratiometric assays, 120 pM mNG-NL calibrator luciferase was included in the LUNAS mix. For RT-RPA-LUNAS reactions, the master mix additionally included 2.11 U/µL SuperScript IV reverse transcriptase (Invitrogen), 0.105 U/µL RNase H (NEB) and 1.05 U/µL murine RNase inhibitor (NEB). This master mix was used to resuspend lyophilized RPA reaction components (TwistAmp Basic kit, TwistDx) at 47.5 µL per pellet. Reactions were performed in a total volume of 20 µL, combining 1 µL sample with 19 µL reaction mixture in a Nunc 384-well non-treated flat-bottom white microplate (Thermo Fisher). Luminescence spectra (413 – 563 nm) were recorded over time at 40°C on a Tecan Spark 10M plate reader with an integration time of 100 ms. The blue/green emission ratio was calculated by dividing luminescence intensity at 458 ± 12.5 nm by the intensity at 518 ± 12.5 nm.

Clinical validation

For testing clinical eSwab (Copan, Italy) samples without RNA isolation, viral lysis and nuclease inactivation was performed by adding an inactivation buffer (200 mM TCEP, 2 mM EDTA, 2 U/µL murine RNase inhibitor, 20 mM Tris-HCl, pH 8.0) to the sample in 1:1 ratio, followed by incubation at 95°C for 5 min. The compatibility of RT-RPA-LUNAS with this sample pretreatment method, based on Arizti-Sanz et al.[12] and Qian et al.[13], was tested using mock eSwab, as well as VTM (HiMedia HiViral medium) and saliva (obtained from a healthy donor) samples, which were prepared by spiking in IVT ORF1a target RNA. Target RNA was added before heating in order to simulate release of RNA from lysed virus before complete denaturation of RNases.

Clinical RNA isolate samples were extracted from nasopharyngeal swabs collected in either 1 mL eSwab or 2 mL PurePrep TL+ buffer (MolGen). For eSwabs, 150 µL sample was combined with 150 µL MP96 external lysis buffer (Roche) for viral lysis. RNA was extracted from 300 µL of this mixture or from 300 µL of samples collected in PurePrep TL+ directly using the STARMag 96x4 Viral DNA/RNA Universal kit (SeeGene, South-Korea) on a Hamilton Starlet, and was eluted in 100 µL.

The one-pot ratiometric RT-RPA-LUNAS assay was prepared as described above, with a higher calibrator luciferase (24 pM final concentration) and NanoGlo substrate concentration (400-fold final dilution). One or two NTC reactions (water input) were included per assay run of 18 – 56 duplicate reactions. Samples (1 µL) were added to reaction mixtures (19 µL) in a 96-well white PCR plate (VWR) on ice, which was then briefly centrifuged in a lettuce spinner[14] before placing it in a heating block set at 40°C (monitored by external thermometer) within a black EPP box to exclude ambient light. Luminescence was recorded using a Sony DSC-RX100 III digital camera fitted through a hole in the lid of the box, with 30s exposure time, f/1.8 and ISO-6400. The camera was controlled by the Sony Imaging Edge Mobile app on an Android device with an auto clicker app (Click Assistant - Auto Clicker by Y.C. Studio) for continuous shooting over 1 hour. Resulting RAW images were converted to 16 bit TIFF files using Sony Imaging Edge Desktop, and mean blue (B) and green (G) intensities per well were extracted from split RGB channels in ImageJ (v1.53q). Reactions were regarded as positive for SARS-CoV-2 if the blue/green ratio ($BGR(t_i)$) exceeded a threshold value $BGR_T(t_i) = BGR_{smoothNTC}(t_i) + 6 \times SD_{All,1\text{-}3min}$ for t_i to t_{i+2}, with $BGR_{smoothNTC}(t_i)$ the moving average blue/green ratio of the NTC over (t_{i-5}, t_{i+5}), and $SD_{All,1\text{-}3min}$ the standard deviation of the blue/green ratio of all reactions between t = 1 min and t = 3 min. The reported LUNAS threshold time t_T equals the first t_i satisfying the condition $BGR(t_i) > BGR_T(t_i)$ for (t_i, t_{i+2}).

RT-qPCR and ddPCR testing of clinical samples

RNA was extracted as described above, followed by RT-PCR using the Allplex SARS-CoV-2 assay (SeeGene, South-Korea) on a CFX96 thermocycler (Biorad), simultaneously detecting four different target genes: E, N, RdRp and S (the latter two are combined in one fluorescent signal). Data was analyzed with the SARS-CoV-2 Viewer software (SeeGene). Some samples were initially tested using the BioFire Respiratory Panel 2.1+ (bioMérieux, France), enabling qualitative detection of multiple pathogens.

To quantify absolute ORF1a_1 RT-RPA-LUNAS target concentrations and correlate these with RT-qPCR C_t values, droplet-digital PCR was performed for 16 extracted RNA samples using the 1-Step RT-ddPCR Advanced Kit for Probes (BioRad) and the CFX96 thermocycler (BioRad) and QX200 ddPCR system (BioRad). Three different reactions were performed per sample, targeting the E- and the N-gene as well as the ORF1a_1 region targeted by the RT-RPA-LUNAS assay. Samples were diluted 1 – 1000-fold to avoid overloading and 5 µL of diluted sample was added per 22 µL PCR reaction. Data was analysed using BioRad QX One (v1.2) and linear regression of the correlation between resulting concentrations and RT-qPCR C_t values was performed in Origin 2020 (OriginLab). All results are listed in Table S2.

Thermodynamic model

To shed light on the dependence of the LUNAS response on the various thermodynamic parameters involved, we developed a model of the system (Figure S1). In this model, the total concentration of the sensor RNP complexes was divided over an active (i.e. DNA-binding competent) and an inactive fraction in a 25:75 ratio, in accordance with previous reports[15,16]. The DNA-binding incompetent sensor complexes (denoted 'Ld' and 'Sd' for the LB and SB complexes respectively) were still included in the model, to account for background signal derived from the split NanoLuc parts of these complexes, but conversion to active complexes (denoted 'La' and 'Sa') and vice versa was excluded. For the interaction of dCas9-SB:gRNA_A and dCas9-LB:gRNA_B with the target DNA (denoted 'T') the same single dissociation equilibrium constant was defined ('K_DC'), assuming differences in affinity depending on the exact gRNA/protospacer sequence to be small for functional LUNAS RNP complex pairs. Moreover, dCas9-SB:gRNA_A and dCas9-LB:gRNA_B are considered to bind the target DNA in a non-cooperative fashion, hence dCas9-SB:gRNA_A binds to free target DNA with the same affinity as to target DNA already bound by dCas9-LB:gRNA_B (denoted 'LaT'). Upon formation of the ternary 'LaSaT' complex, the high local concentration (the effective molarity (EM)) of the split NanoLuc fragments promotes complementation, transitioning to the luminescent 'LaSaTa' complex. As non-templated split NanoLuc complementation could also result in luminescence, the total luminescent signal is modelled as the sum of the concentrations of LaSaTa, LaSa, LdSd, LdSa and LaSd, multiplied by a constant.

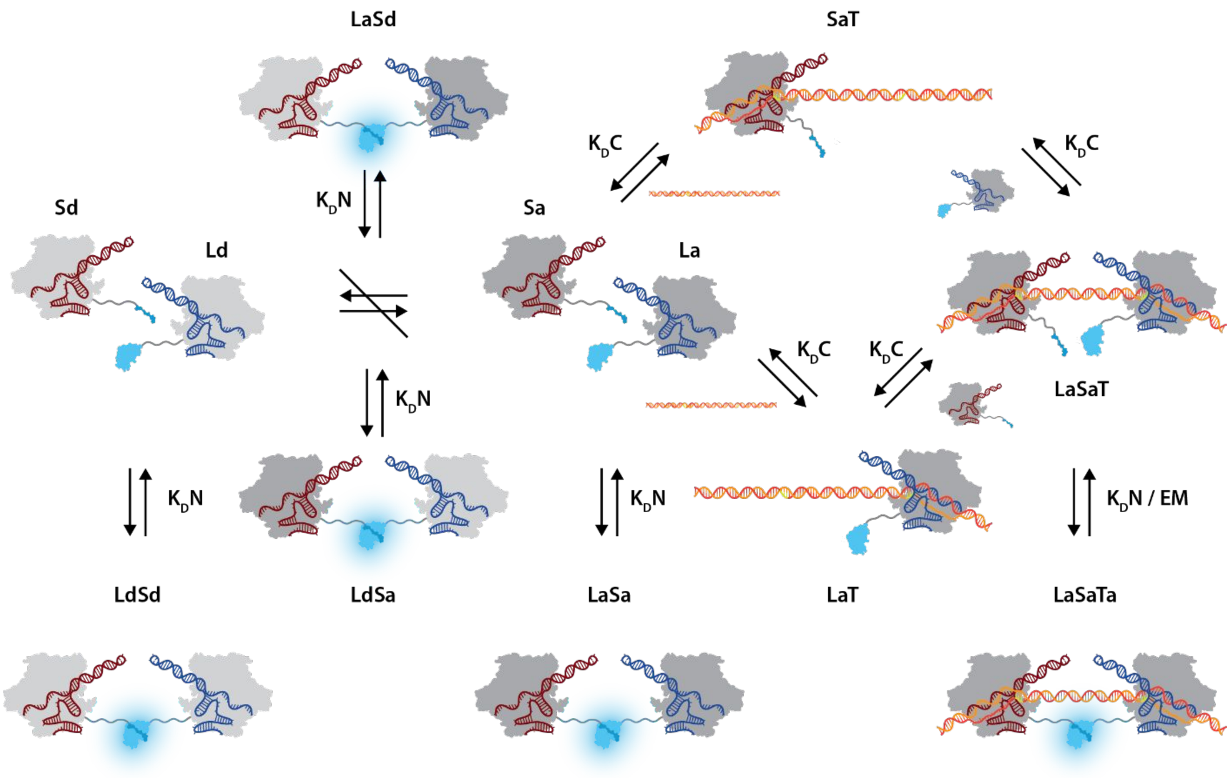

Figure S1: Model of the thermodynamic interactions in the LUNAS assay.

The model was implemented using a general framework for equilibrium models developed by Geertjens *et al.*[17], using the following input to build the model in the Python tool (with K_DN = `KDNanoBiT` and K_DC = `KDdCas9`):

```
Equations:

Ld+Sd = LdSd; KDNanoBiT
Ld+Sa = LdSa; KDNanoBiT
La+Sd = LaSd; KDNanoBiT
La+Sa = LaSa; KDNanoBiT
La+T = LaT; KDdCas9
Sa+T = SaT; KDdCas9
LaT+Sa = LaSaT; KDdCas9
SaT+La = LaSaT; KDdCas9
LaSaT = LaSaTa; KDNanoBiT/EM

data_mode: custom
custom_input: constant * (LaSaTa + LaSa + LdSd + LdSa + LaSd)
```

From this, a model was generated in the framework, which is further detailed below for completeness:

The equilibrium concentrations of the dependent species can be determined based on the concentrations of the independent species and the corresponding equilibrium constant, using the following relations:

```
LdSd: Ld*Sd/KDNanoBiT
LdSa: Ld*Sa/KDNanoBiT
LaSd: La*Sd/KDNanoBiT
LaSa: La*Sa/KDNanoBiT
```

```
LaT: La*T/KDdCas9
SaT: Sa*T/KDdCas9
LaSaT: La*Sa*T/KDdCas9**2
LaSaTa: EM*La*Sa*T/(KDNanoBiT*KDdCas9**2)
```

The mass balance of the independent species in terms of free and complexed forms:

```
Sa_tot: LaSa + LaSaT + LaSaTa + LdSa + Sa + SaT
La_tot: La + LaSa + LaSaT + LaSaTa + LaSd + LaT
T_tot: LaSaT + LaSaTa + LaT + SaT + T
Sd_tot: LaSd + LdSd + Sd
Ld_tot: Ld + LdSa + LdSd
```

Substituting the relations above in the mass balance equations yields:

```
Sa_tot = EM*La*Sa*T/(KDNanoBiT*KDdCas9**2) + Sa  + Sa*T/KDdCas9 +
La*Sa*T/KDdCas9**2 + La*Sa/KDNanoBiT + Ld*Sa/KDNanoBiT

La_tot = EM*La*Sa*T/(KDNanoBiT*KDdCas9**2) + La  + La*T/KDdCas9 +
La*Sa*T/KDdCas9**2 + La*Sa/KDNanoBiT + La*Sd/KDNanoBiT

T_tot = EM*La*Sa*T/(KDNanoBiT*KDdCas9**2) + T  + La*T/KDdCas9 +
Sa*T/KDdCas9 + La*Sa*T/KDdCas9**2

Sd_tot = Sd  + La*Sd/KDNanoBiT + Ld*Sd/KDNanoBiT

Ld_tot = Ld  + Ld*Sa/KDNanoBiT + Ld*Sd/KDNanoBiT
```

Finally, these equations were rewritten to equal zero, and divided by the total concentrations on both sides in order to reach a solution faster during solving:

```
Sa: (EM*La*Sa*T/(KDNanoBiT*KDdCas9**2) + Sa - Sa_tot + Sa*T/KDdCas9 +
La*Sa*T/KDdCas9**2 + La*Sa/KDNanoBiT + Ld*Sa/KDNanoBiT) / Sa_tot = 0

La: (EM*La*Sa*T/(KDNanoBiT*KDdCas9**2) + La - La_tot + La*T/KDdCas9 +
La*Sa*T/KDdCas9**2 + La*Sa/KDNanoBiT + La*Sd/KDNanoBiT) / La_tot = 0

T: (EM*La*Sa*T/(KDNanoBiT*KDdCas9**2) + T - T_tot + La*T/KDdCas9 +
Sa*T/KDdCas9 + La*Sa*T/KDdCas9**2) / T_tot = 0

Sd: (Sd - Sd_tot + La*Sd/KDNanoBiT + Ld*Sd/KDNanoBiT) / Sd_tot = 0

Ld: (Ld - Ld_tot + Ld*Sa/KDNanoBiT + Ld*Sd/KDNanoBiT) / Ld_tot = 0
```

This model was fitted to the combined data presented in Figure 2C using ‘KDNanoBiT’ = 2.5E-6 as known parameter[18]. For dCas9:gRNA complex binding to target DNA, an upper limit in K_D of ~0.5 nM was previously reported based on EMSA experiments[19], and here 0.1 nM was taken as an initial value for ‘KDdCas9’. The effective molarity of the split NanoLuc fragments (EM) depends on the length and flexibility of the linkers connecting them to the dCas9 proteins, as well as the distance that has to be bridged by the linkers for luciferase complementation. An initial value for the EM was estimated based on the wormlike chain model[20,21]. Considering the 21 residue linkers are made up mostly of GGS repeats, a persistence length of 3.7 Å was assumed[21]. Based on the structure of DNA bound dCas9:gRNA (PDB: 5F9R[22]) and that of NanoLuc (PDB: 5IBO), the distance to be bridged between anchor points (dCas9 C-terminus and SB/LB N-terminus) for the 50 bp interspace target used is roughly 49 Å. From this, we estimated an EM of ~ 31 µM[20]. For the factor converting luminescent entity concentrations to luminescence signal intensity, the initial value was set as ‘constant’ = 1E+15. The following parameter estimates were obtained (fitted lines shown in Figure 2C):

```
EM = 1.048e-05
KDdCas9 = 1.767e-11
constant = 1.939e+15

Root Mean Squared Error (10 nM dCas9-SB RNP + 1 nM dCas9-LB RNP) = 2.01e+04
Root Mean Squared Error (1 nM dCas9-SB RNP + 1 nM dCas9-LB RNP) = 2.02e+04
R² (10 nM dCas9-SB RNP + 1 nM dCas9-LB RNP)= 0,982
R² (1 nM dCas9-SB RNP + 1 nM dCas9-LB RNP)= 0,955
```

To gauge the dependence of the signal response on tunable sensor parameters, we performed simulations in which the concentration ratio of sensor RNP complexes was varied, and we simulated the effect of different split NanoLuc binding affinities (Figure S2). For this, parameter estimates described above were used.

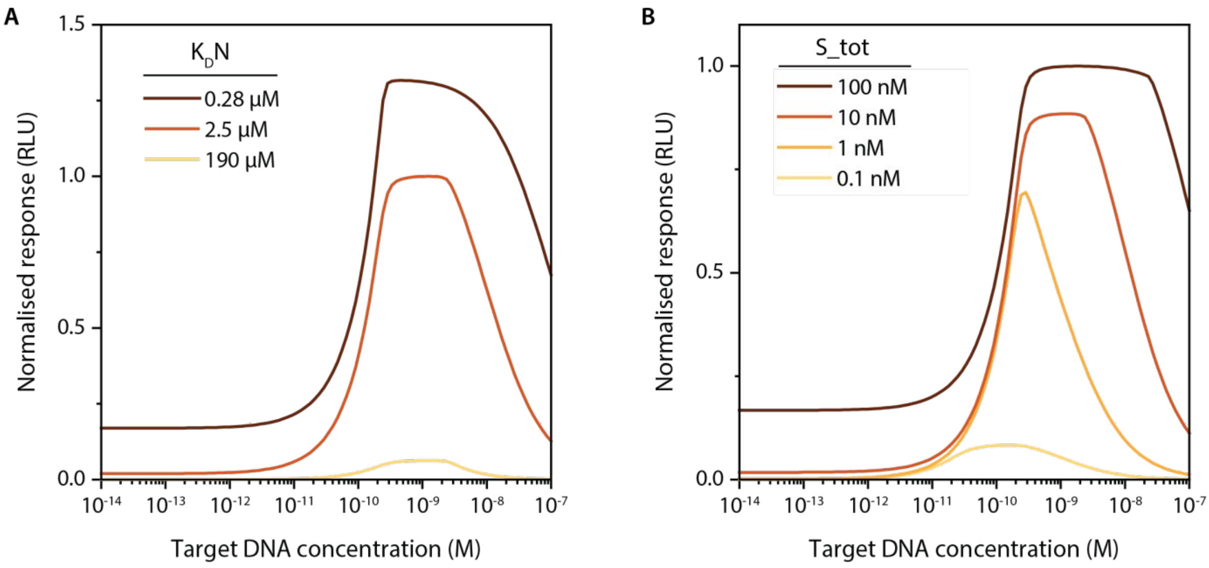

Figure S2: Model simulations of LUNAS response for a variation in sensor parameters. A Varying the affinity of small BiT (SB) for binding to large BiT (LB) (K_DN). **B** Varying the total concentration of dCas9-SB:gRNA_A (S_tot), keeping total dCas9-LB:gRNA_B at 1 nM. For both **(A)** and **(B)**, the response was normalised to the maximum of the orange line, corresponding to K_DN = 2.5 μM and S_tot = 10 nM. Other parameters were set as follows: EM = 10.48 μM, K_dC = 17.67 pM, constant = 1.939×10^{15}. For **(B)**, K_DN = 2.5 μM. For **(A)**, L_tot = 1 nM and S_tot = 10 nM.

Ethics statement

The use of anonymised clinical samples for this study was evaluated and approved by the local medical ethics review committee of the Rijnstate Hospital (reference number: KCHL 2021-1950), and samples were acquired in accordance with the Declaration of Helsinki. The samples used here were obtained as part of standard clinical COVID-19 testing and patients did not object to the use of remnant sample material for quality purposes and research.

Safety statement

No unexpected or unusually high safety hazards were encountered.

Figures

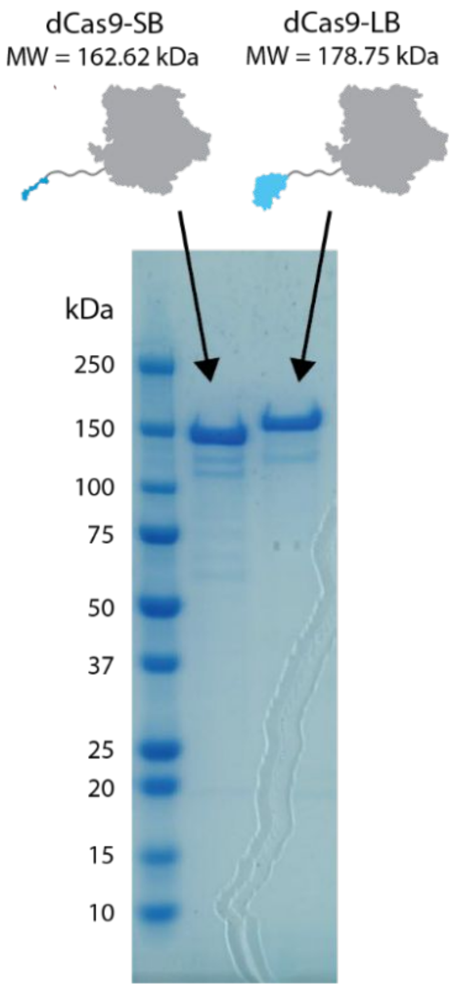

Figure S3: Expression and purification of LUNAS proteins. Reducing SDS-PAGE (4-20%) analysis of dCas9-SB and dCas9-LB. After expression in E. coli BL21, the proteins were purified by Strep-Tactin XT chromatography.

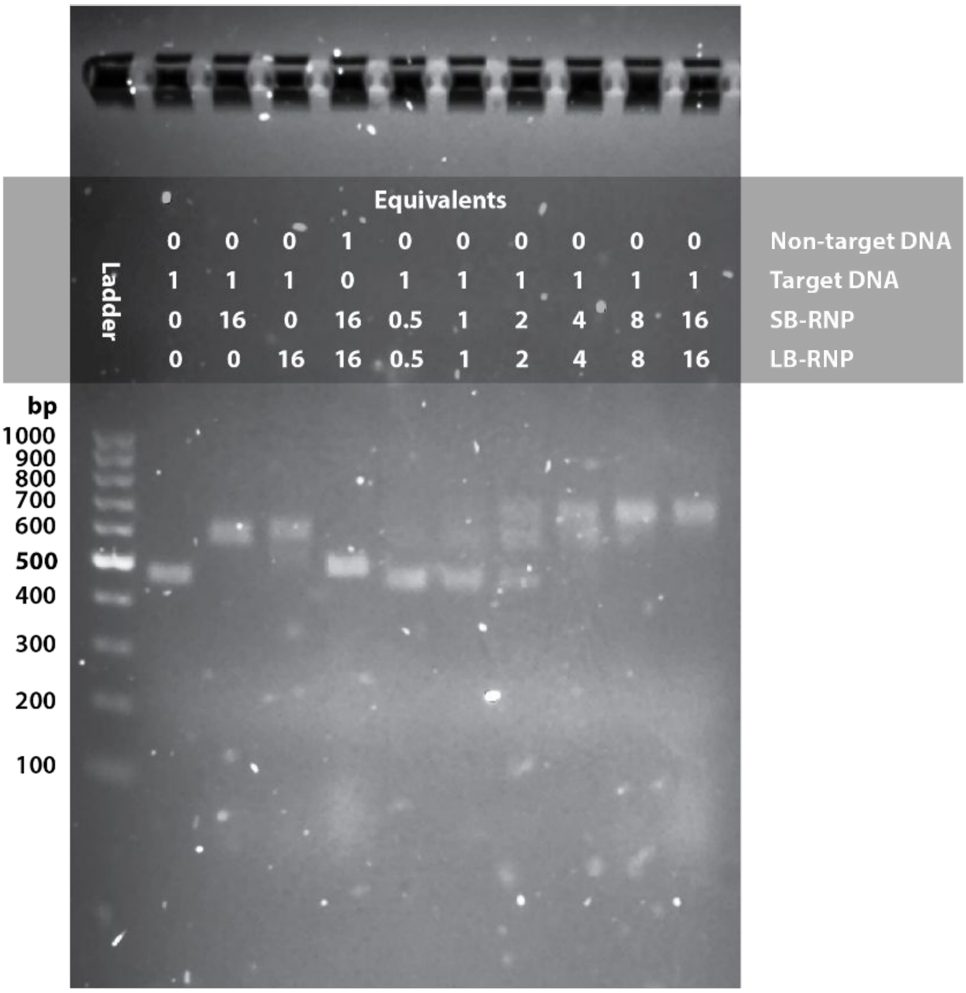

Figure S4: Electrophoretic mobility shift assay (EMSA) with LUNAS RNPs. 12.5 nM of non-target (510 bp) or target (473 bp, 30 bp interspace) dsDNA fragment was incubated with 0.5 to 16 equivalents of RNP-SB and/or RNP-LB for 1 hour at RT in LUNAS RNP buffer. Reactions were loaded on a 2% agarose gel containing 1x SYBR safe and run for 55 minutes at 100V in 1x TAE buffer.

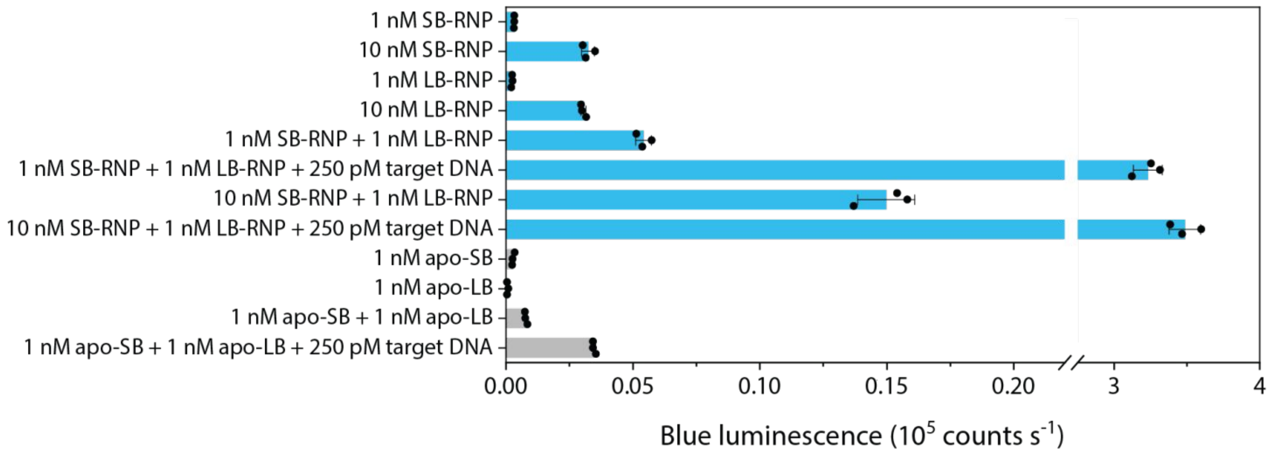

Figure S5: LUNAS background luminescence. Individual RNPs show low levels of luminescence, and the background signal observed in LUNAS assays appears to be mostly resulting from split-NanoLuc complementation upon target-independent binding of SB-RNP and LB-RNP to each other. Using 10 nM SB-RNP instead of 1 nM SB-RNP results in roughly 3-fold higher background signal. Apo proteins (i.e. without gRNA) also show low luminescence, which does not increase above LUNAS background levels upon presence of DNA, confirming that gRNA-guided specific target binding is required for strong increase in luminescence. Bars represent means, error bars show SD and individual replicates (n = 3) are represented as dots.

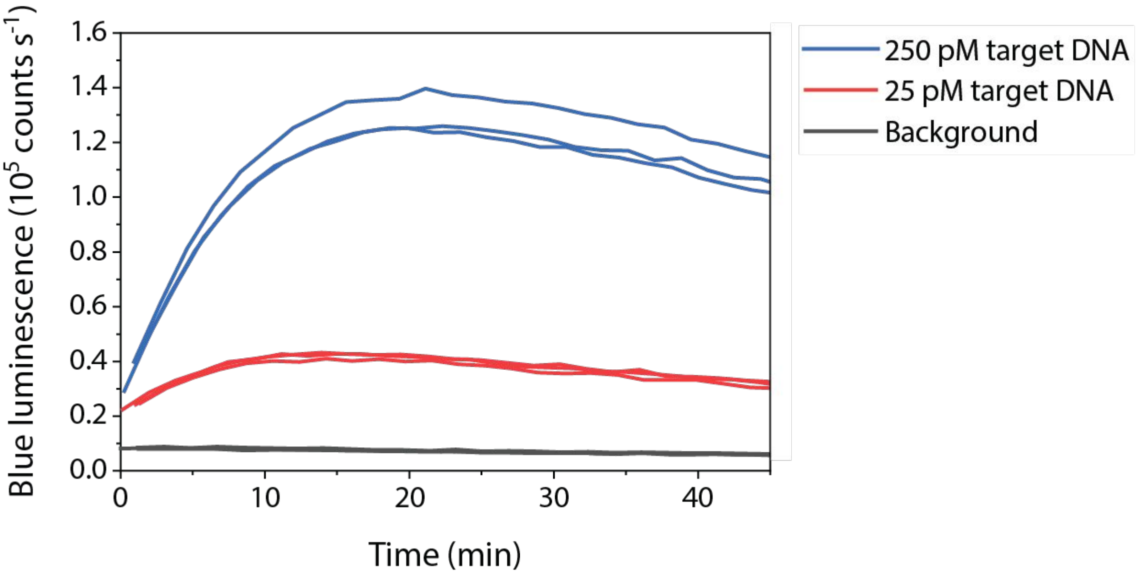

Figure S6: Kinetics of intensiometric LUNAS. 1 nM dCas9-SB:gRNA_T7A and 1 nM dCas9-LB:gRNA_T7B were combined with target (30 bp interspace) and NanoGlo substrate (1000-fold final dilution) directly before start of measurement over time. Individual replicate traces (n = 3) are shown.

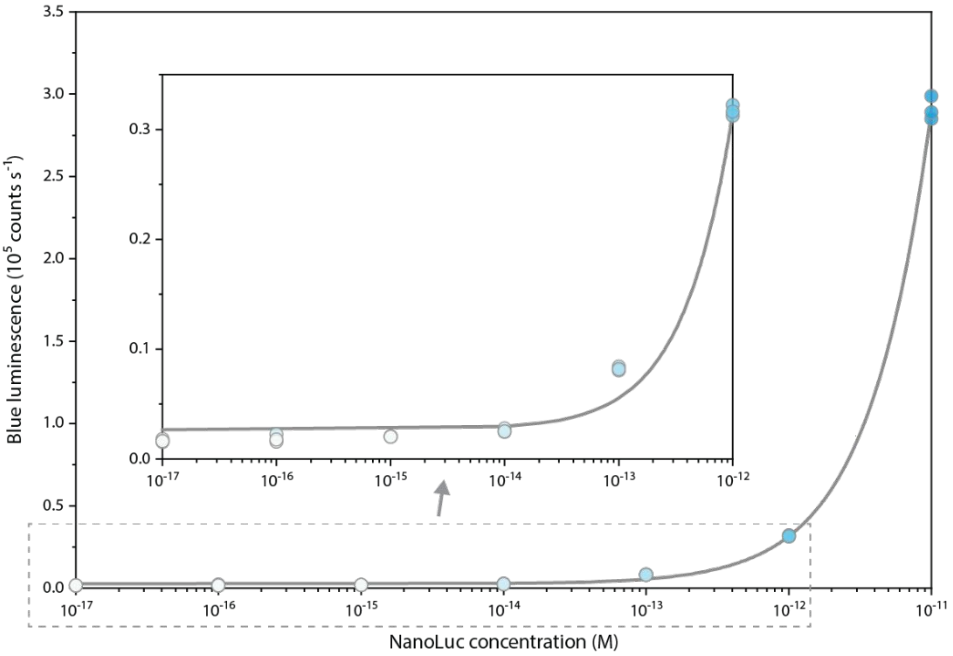

Figure S7: NanoLuc titration curve. A 10-fold serial dilution series of NanoLuc in LUNAS RNP buffer was combined with NanoGlo substrate (2000-fold final dilution) and blue luminescence intensity was measured. The inset zooms in on the portion of the main graph indicated in the dashed box. This data shows that the minimal active NanoLuc concentration that can be detected under LUNAS conditions in 20 µL is in the 10 – 100 fM range. Since complemented split-NanoLuc (NanoBiT) has a relative luciferase activity of ~ 37% compared to that of full-length NanoLuc, the minimal concentration of complemented split-NanoLuc that can be detected under these conditions is presumably on the order of ~100 fM[18]. Individual replicates (*n* = 3) are shown as circles, the line represents a linear fit to the full data range (Pearson's r = 0.99969; R^2 (COD) = 0.99938).

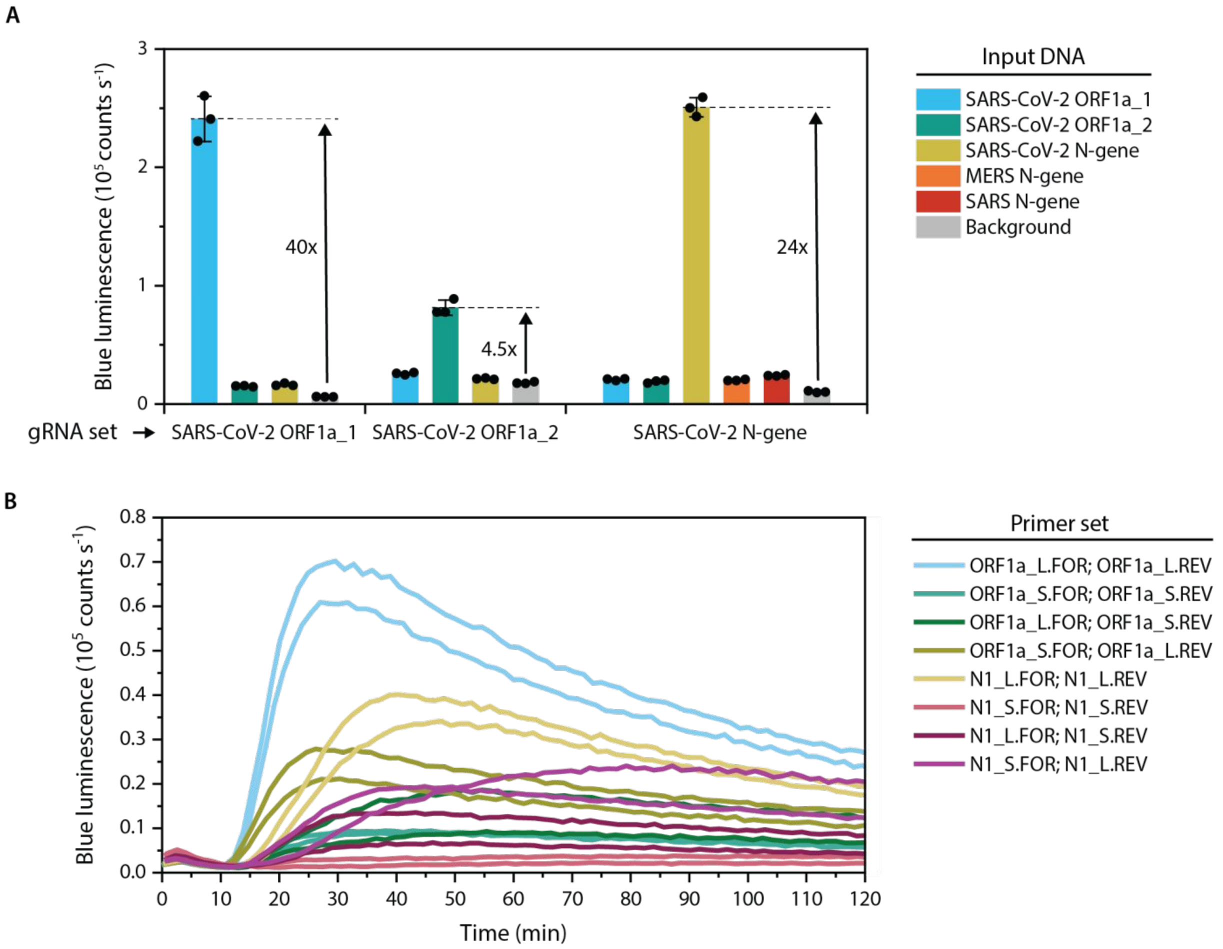

Figure S8: RPA-LUNAS SARS-CoV-2 assay gRNA and primer screening. A Screening 3 pairs of gRNAs for best performance in a LUNAS assay. dCas9-LB and dCas9-SB proteins were complexed with 3 pairs of gRNAs targeting different regions in the SARS-CoV-2 genome (ORF1a_1 and ORF1a_2 designate two different regions of ORF1a, see Table S1). With these dCas9 RNPs (1 nM of both SB and LB), LUNAS assays were performed on matching target cDNA fragments (250 pM), as well as on the non-matching fragments (250 pM) used as non-target controls. Additionally, for the N-gene LUNAS assay, MERS and SARS N-gene cDNA fragments (250 pM) were used as non-target controls featuring strong sequence-homology with the target. Clearly, the ORF1a_1 and N-gene gRNA sets show the best LUNAS performance, with a 40- and 24-fold increase in blue luminescence over background level respectively, while the ORF1a_2 set only shows a moderate 4.5-fold increase and low absolute intensity. The low response of the SARS-CoV-2 N-gene LUNAS for MERS and SARS N-gene cDNA fragments compared to the target SARS-CoV-2 N-gene cDNA fragment demonstrates the assay specificity. Bars represent means of technical replicates (n = 3), which are indicated as black dots. Error bars show SD. **B** Screening RPA primer sets for best performance in an RPA-LUNAS assay. Two forward and reverse primers were designed for combination with the two best performing SARS-CoV-2 LUNAS assays in **(A),** one partly complementary to the PAM-distal protospacer sequence ('_S' primers), the other only binding to the sequence 3' from the protospacer ('_L' primers). All 4 combinations of primers per target region were tested. For both the N and ORF1a target region, the assays using the combination of only '_L' primers showed the highest signal. Clearly, the ORF1a assay performed best, showing the quickest onset of signal increase, and reaching the highest absolute intensity. The assays were performed at 42°C, using 200 cp of input cDNA fragment. Individual replicate traces are shown (n = 2).

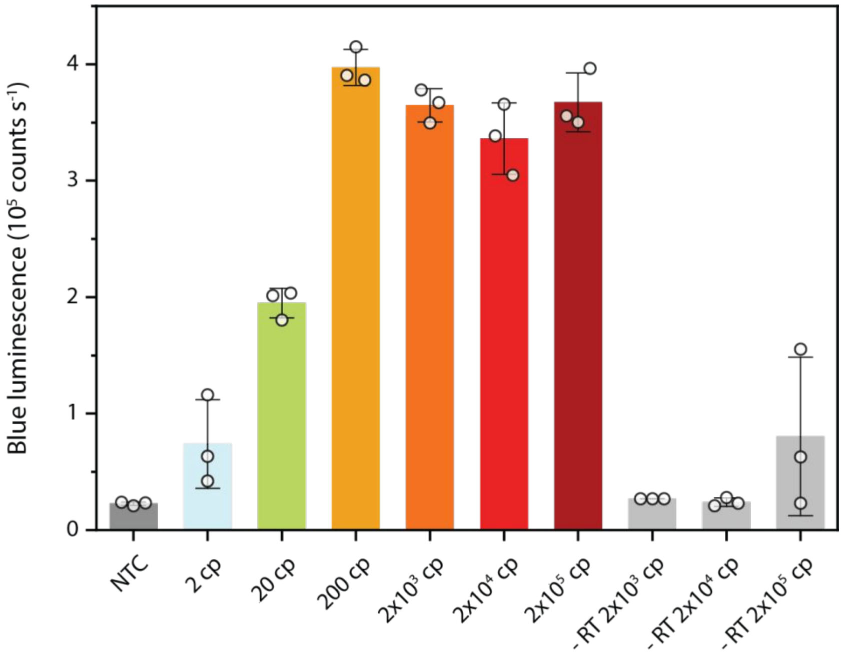

Figure S9: 2-step RT-RPA-LUNAS SARS-CoV-2 assay. RT-RPA reactions were performed for a range of inputs of IVT SARS-CoV-2 ORF1a RNA fragment. Additionally, control reactions in the absence of the reverse transcriptase (labelled '- RT') were performed to verify successful DNase I-based degradation of IVT template DNA. The triplicate RPA reactions were added to LUNAS assay reactions and resulting blue luminescence was measured. For the reactions without RT, only for an input of $2x10^5$ cp a LUNAS response is observed for 2 of 3 replicates, similar to the response for 2 cp input in reactions including RT, confirming that the RT-RPA-LUNAS assay indeed detects RNA and that there is virtually no contribution of remainder IVT template DNA to the observed results. Bars represent means of technical replicates (n = 3), which are indicated as circles. Error bars show SD.

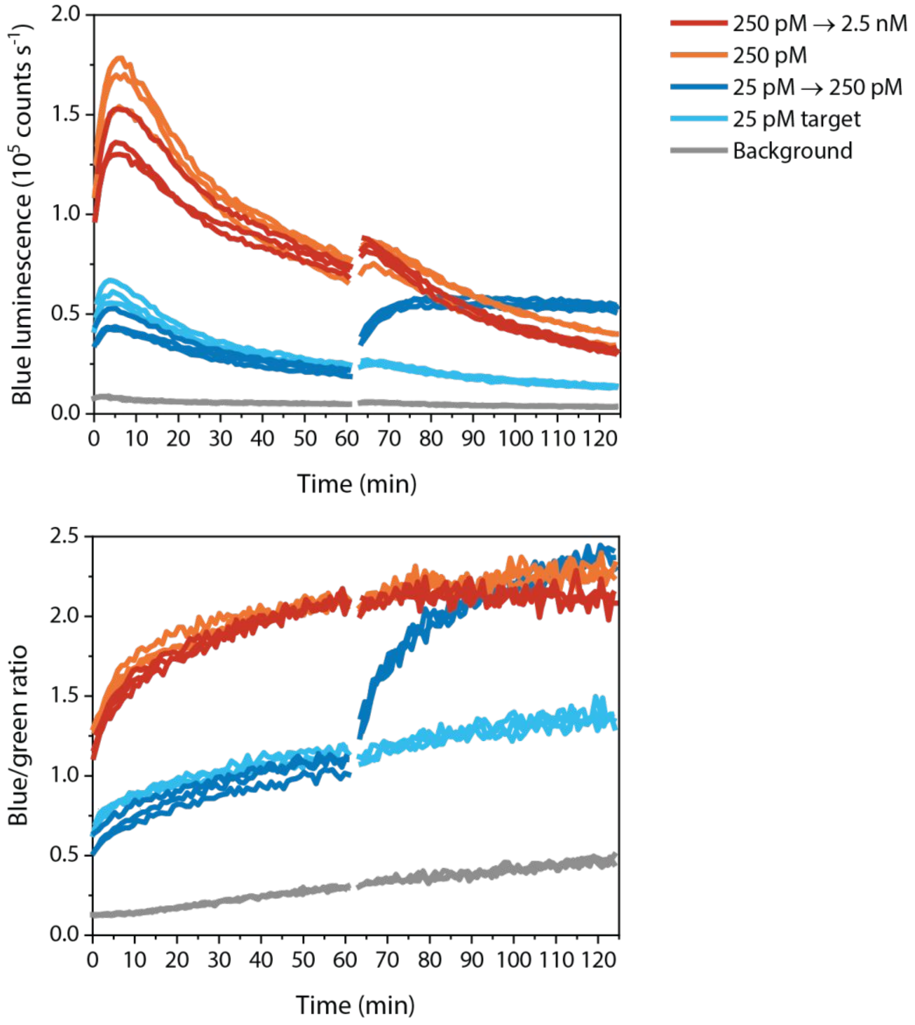

Figure S10: LUNAS kinetics upon increase in target concentration following initial equilibration. dCas9-SB:T7A (1 nM) and dCas9-LB:T7B (1 nM) were incubated with target DNA (30 bp interspace) at indicated concentrations for 1 h, after which the target concentration was increased 10-fold in part of the reactions (red and dark blue lines). The top panel shows the resulting blue luminescence intensity as measured over time. The mNG-NL calibrator luciferase was also included in the reactions (see main text), and the bottom panel shows the blue/green ratio over time. While an increase in target concentration from 25 pM to 250 pM can be seen to result in a rapid increase in blue signal, the increase from 250 pM to 2.5 nM does not result in substantial change in signal compared to that of the control left at 250 pM. This result confirms the extremely slow dissociation rate of dCas9 RNPs, as quick dissociation and redistribution of the dCas9 RNPs would result in a lower signal for 2.5 nM compared to 250 pM target (see Figure 2C). LUNAS assays were prepared as described for 1-pot RPA-LUNAS assays, excluding primers to preclude RPA, and were performed at 40°C to resemble RPA-LUNAS conditions. Individual replicate traces are shown (n = 3).

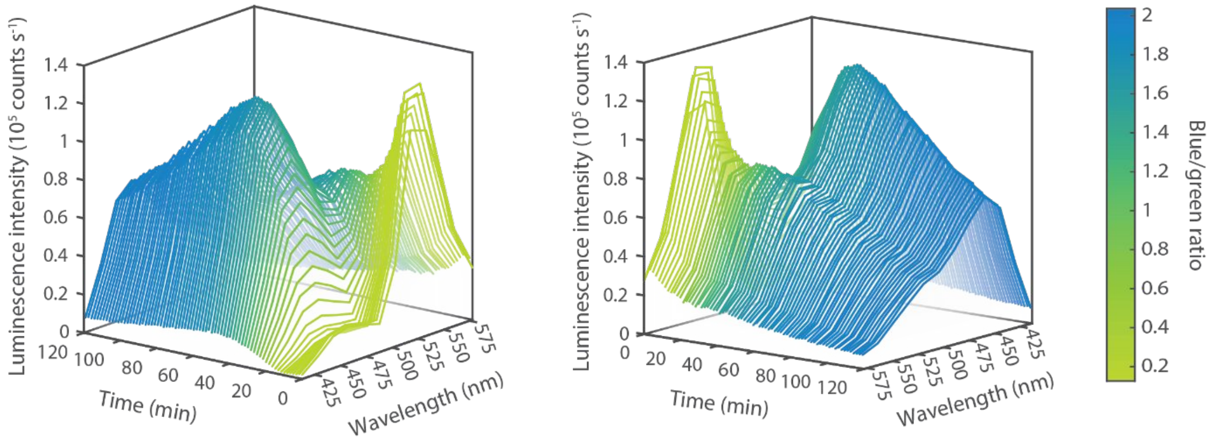

Figure S11: Ratiometric RPA-LUNAS luminescence spectra over time. Luminescence spectra of a single SARS-CoV-2 RT-RPA-LUNAS reaction replicate (from Figure 4D, 200 cp input) as measured over time (extended data version of Figure 4C). The right waterfall graph shows the backside of the left graph. The rapid increase in blue signal can be observed, followed by an overall gradually decreasing luminescence due to substrate depletion, which can be observed for the green signal already from the start. However, the blue/green ratio stays relatively constant over time after the initial rise in blue LUNAS signal.

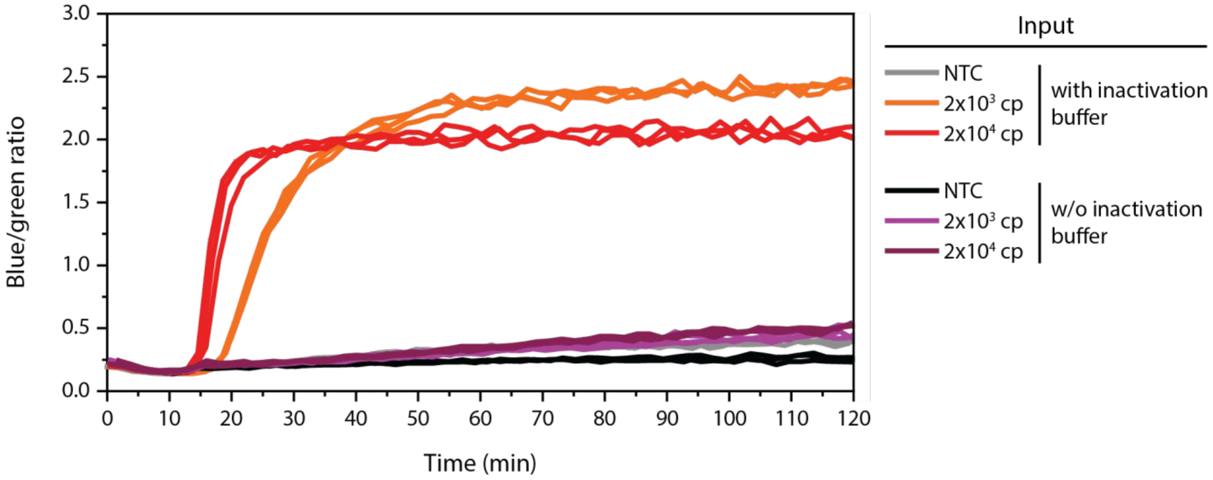

Figure S12: Importance of RNase inactivation for RT-RPA-LUNAS in saliva samples. The 1-pot RT-RPA-LUNAS SARS-CoV-2 assay was performed for mock Covid-19 saliva samples pretreated with or without the RNase inactivation buffer. Both pure saliva and saliva 1:1 diluted in inactivation buffer (100 mM TCEP, 1mM EDTA, 1U/µL murine RNase inhibitor, 10 mM Tris-HCl, pH 8.0) were spiked with IVT SARS-CoV-2 target RNA fragment and then heated for 5 minutes at 95°C. After a brief cool-down, these samples were added to ratiometric RT-RPA-LUNAS reactions. Clearly, only the samples treated with the inactivation buffer show a clear LUNAS response. Individual replicate traces are shown (*n* = 3).

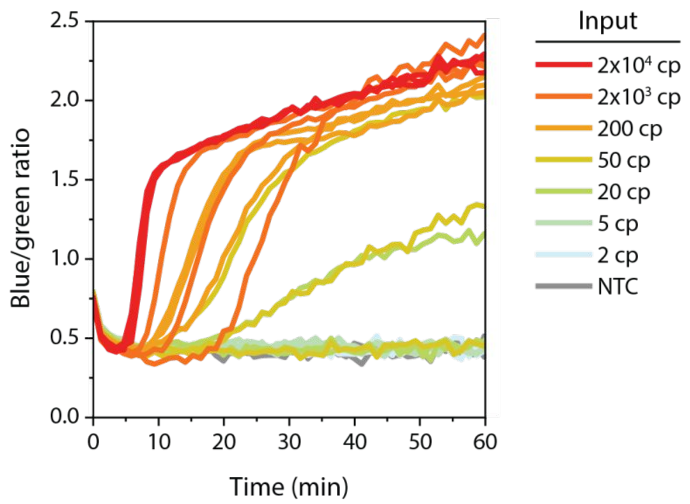

Figure S13: Camera-based RT-RPA-LUNAS for SARS-CoV-2 RNA detection from saliva. Camera-based readout of experiment that is similar to the one shown in Figure 4F with saliva input, with ratiometric RT-RPA-LUNAS response extracted from pictures recorded by camera. Individual replicate traces (*n* = 3) are shown.

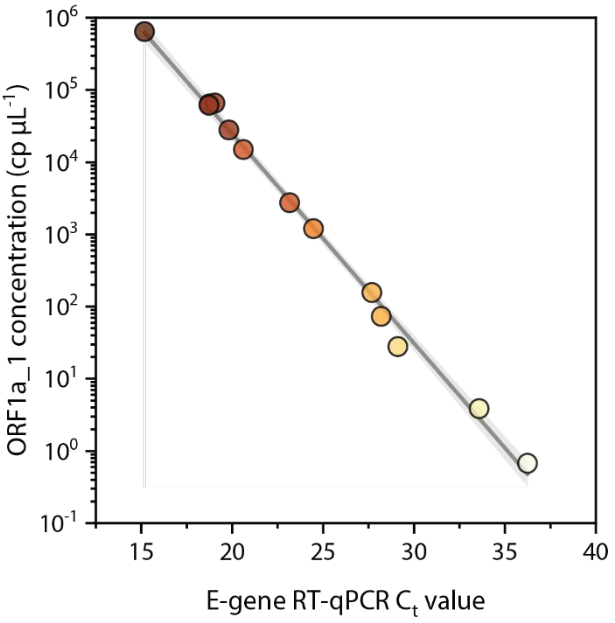

Equation	$\log_{10}(y) = a + b*x$
a	10.19528 ± 0.14663
b	-0.29009 ± 0.00587
Residual Sum of Squares	0.18477
Pearson's r	-0.99775
R-Square (COD)	0.99551
Adj. R-Square	0.9951

Figure S14: RT-ddPCR quantification of SARS-CoV-2 RNA in clinical samples. ORF1a_1 concentration quantitated by ddPCR versus corresponding E-gene RT-qPCR C_t value. A linear regression model was fitted to the log-transformed data (grey line, with 95% confidence bands), parameters are shown in the table on the right. Single replicates were measured and are represented as filled circles. The reported concentrations correspond to the RNA concentrations in the extraction eluates.

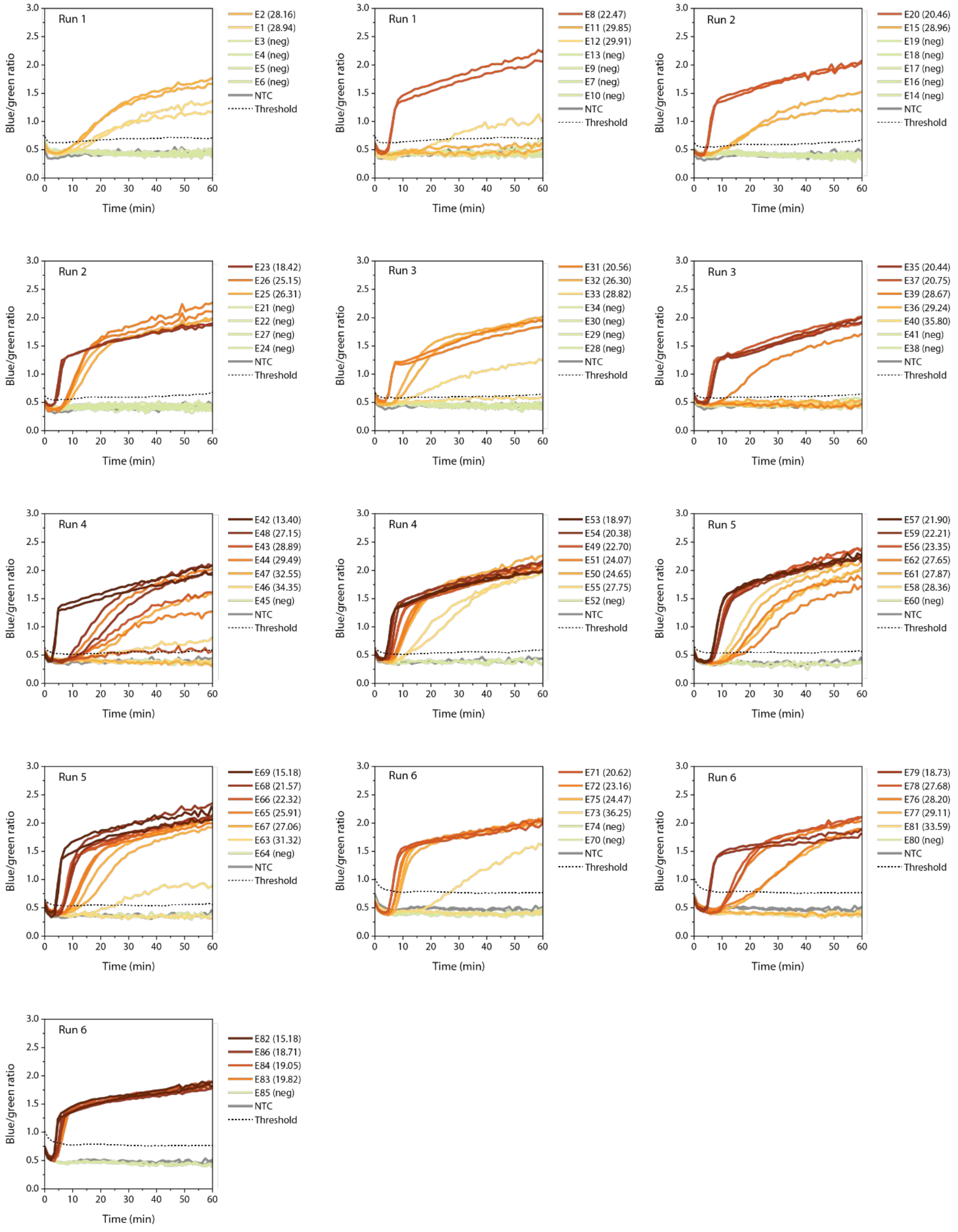

Figure S15: RT-RPA-LUNAS ratiometric response traces of all extracted clinical samples. Traces are grouped in panels per assay run, with data divided over multiple panels per run for clarity. In the legend, the sample ID is followed by the corresponding E-gene RT-qPCR C_t value in brackets.

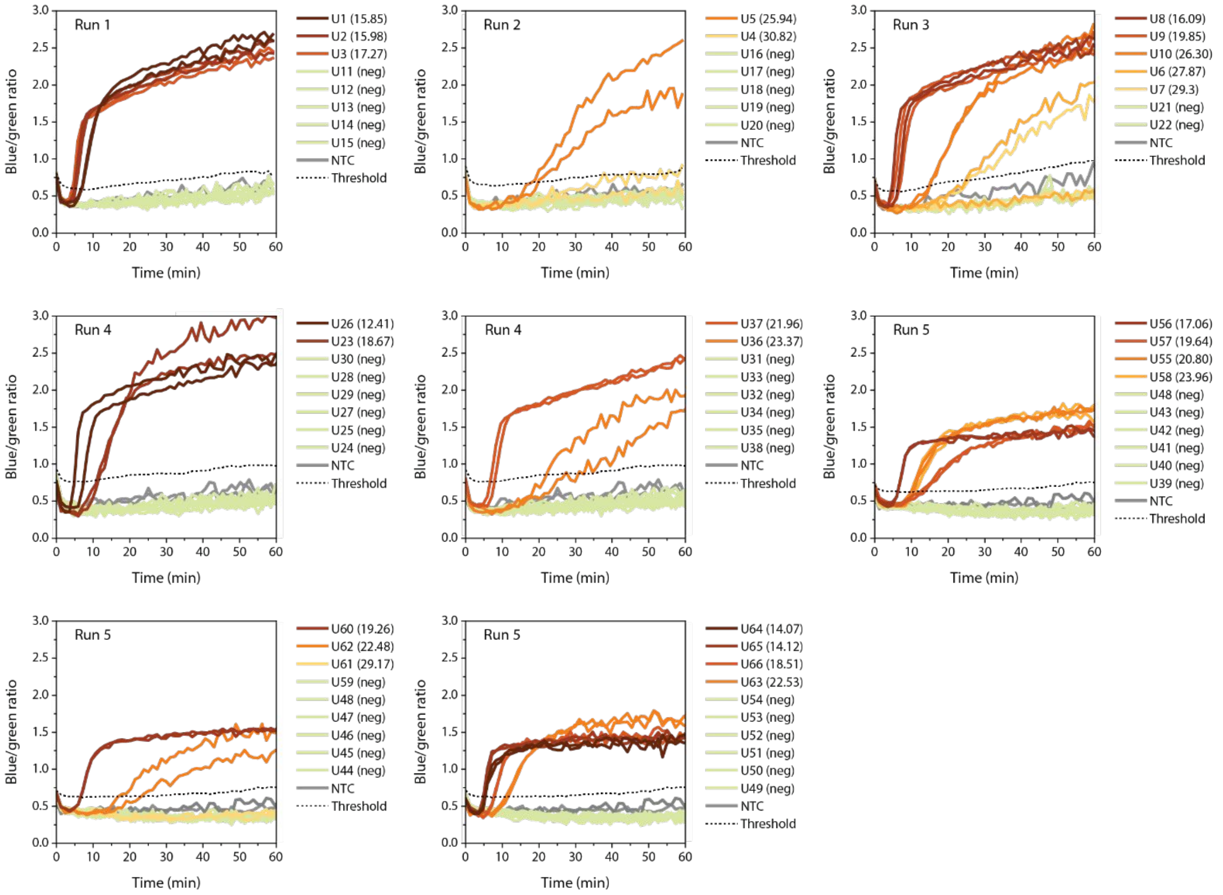

Figure S16: RT-RPA-LUNAS ratiometric response traces of all unextracted clinical samples. Traces are grouped in panels per assay run, with data for some runs divided over multiple panels for clarity. In the legend, the sample ID is followed by the corresponding E-gene RT-qPCR C_t value in brackets.

Tables

Table S3: Comparison of RT-RPA-LUNAS SARS-CoV-2 assay performance with that of other recent CRISPR diagnostic methods applied to SARS-CoV-2 detection.

Method	Reference	Steps	Readout	Detection time	Approximate LOD
RT-RPA-LUNAS	This work	1-pot	Bioluminescent	10 – 30 min	200 cp/µL (nasopharyngeal samples, heat-inactivated)
SHINE (SHERLOCK based, RPA + Cas13)	Arizti-Sanz et al.[12]	1-pot	Fluorescent or LFA	40 min - 1 hour	100 - 1000 cp/µL (clinical nasopharyngeal samples, heat inactivated)<br>10 cp/µL (synthetic target)
SHINE v.2 (SHERLOCK based, RPA + Cas13)	Arizti-Sanz et al.[23]	1-pot	Fluorescent or LFA	< 90 min.	200 cp/µL (clinical nasopharyngeal samples, chemically lysed)
DETECTR (LAMP + Cas12)	Broughton et al.[24]	2-step	Fluorescent or LFA	30 - 40 min	10 cp/µL (synthetic target), validated for extracted clinical swab samples
SCOPE (LAMP + Type III CRISPR (TtCmr) + CARF-RNase)	Steens et al.[25]	1-pot / 2-step	Fluorescent	2-step: 35 min<br>1-pot: 180 min	2-step: 25 cp/µL (synthetic target)<br>1-pot: 482 cp/µL (synthetic target)
STOP Covid (SHERLOCK based, RPA + Cas13	Joung et al.[26]	1-pot	Fluorescent	15 – 45 min	33 cp/mL (with extreme concentration of RNA from clinical samples during magnetic bead isolation)
DISCoVER (LAMP + Cas13)	Chandrasekaran et al.[27]	1-pot	Fluorescent	60 min	40 cp/µL (viral stocks in saliva, lysed in integrated device)
sPAMC (RPA + Cas12a)	Lu et al.[28]	1-pot	Fluorescent	15 min	1 - 100 cp/µL (nasopharyngeal samples, heat-inactivated or extracted)

Movie

Movie S1 (separate file). Timelapse of an RT-RPA-LUNAS assay detecting SARS-CoV-2 RNA in clinical samples. Individual photos of one assay with 36 reactions (17 samples plus 1 NTC, in duplicate, corresponding to Figure S15 Run 6) collected over time were combined in a timelapse video, showing the quick shift in observed luminescence color from green to blue for SARS-CoV-2 positive samples (in red boxes) with lower comparator RT-qPCR Ct values. The reactions with SARS-CoV-2 negative inputs (in white boxes) remain green like the NTC (in orange box), as do some of the reactions with SARS-CoV-2 positive inputs having higher comparator RT-qPCR Ct values (≥ 29).

Protein coding sequences and translations

dCas9-SB / dCas9-LB

The following combined sequence codes for dCas9-SB (default) and dCas9-LB (after SpeI restriction digest and subsequent self-ligation of the large fragment). Colour codes: dCas9 highlighted in yellow; SB in cyan; LB in green; SpeI restriction site in magenta, Strep-tag II in red font.

```
atggataagaaatactcaataggcttagctatcggcacaaatagcgtcggatgggcggtg
 M  D  K  K  Y  S  I  G  L  A  I  G  T  N  S  V  G  W  A  V
atcactgatgaatataaggttccgtctaaaaagttcaaggttctgggaaatacagaccgc
 I  T  D  E  Y  K  V  P  S  K  K  F  K  V  L  G  N  T  D  R
cacagtatcaaaaaaaatcttataggggctcttttatttgacagtggagagacagcggaa
 H  S  I  K  K  N  L  I  G  A  L  L  F  D  S  G  E  T  A  E
gcgactcgtctcaaacggacagctcgtagaaggtatacacgtcggaagaatcgtatttgt
 A  T  R  L  K  R  T  A  R  R  R  Y  T  R  R  K  N  R  I  C
tatctacaggagattttttcaaatgagatggcgaaagtagatgatagtttctttcatcga
 Y  L  Q  E  I  F  S  N  E  M  A  K  V  D  D  S  F  F  H  R
cttgaagagtcttttttggtggaagaagacaagaagcatgaacgtcatcctatttttgga
 L  E  E  S  F  L  V  E  E  D  K  K  H  E  R  H  P  I  F  G
aatatagtagatgaagttgcttatcatgagaaatatccaactatctatcatctgcgaaaa
 N  I  V  D  E  V  A  Y  H  E  K  Y  P  T  I  Y  H  L  R  K
aaattggtagattctactgataaagcggatttgcgcttaatctatttggccttagcgcat
 K  L  V  D  S  T  D  K  A  D  L  R  L  I  Y  L  A  L  A  H
atgattaagtttcgtggtcattttttgattgagggagatttaaatcctgataatagtgat
 M  I  K  F  R  G  H  F  L  I  E  G  D  L  N  P  D  N  S  D
gtggacaaactatttatccagttggtacaaacctacaatcaattatttgaagaaaaccct
 V  D  K  L  F  I  Q  L  V  Q  T  Y  N  Q  L  F  E  E  N  P
attaacgcaagtggagtagatgctaaagcgattctttctgcacgattgagtaaatcaaga
 I  N  A  S  G  V  D  A  K  A  I  L  S  A  R  L  S  K  S  R
cgattagaaaatctcattgctcagctccccggtgagaagaaaaatggcttatttgggaat
 R  L  E  N  L  I  A  Q  L  P  G  E  K  K  N  G  L  F  G  N
ctcattgctttgtcattgggtttgacccctaattttaaatcaaattttgatttggcagaa
 L  I  A  L  S  L  G  L  T  P  N  F  K  S  N  F  D  L  A  E
gatgctaaattacagctttcaaaagatacttacgatgatgatttagataatttattggcg
 D  A  K  L  Q  L  S  K  D  T  Y  D  D  D  L  D  N  L  L  A
caaattggagatcaatatgctgatttgtttttggcagctaagaatttatcagatgctatt
 Q  I  G  D  Q  Y  A  D  L  F  L  A  A  K  N  L  S  D  A  I
ttactttcagatatcctaagagtaaatactgaaataactaaggctcccctatcagcttca
 L  L  S  D  I  L  R  V  N  T  E  I  T  K  A  P  L  S  A  S
atgattaaacgctacgatgaacatcatcaagacttgactcttttaaaagctttagttcga
 M  I  K  R  Y  D  E  H  H  Q  D  L  T  L  L  K  A  L  V  R
caacaacttccagaaaagtataaagaaatcttttttgatcaatcaaaaaacggatatgca
 Q  Q  L  P  E  K  Y  K  E  I  F  F  D  Q  S  K  N  G  Y  A
ggttatattgatgggggagctagccaagaagaattttataaatttatcaaaccaatttta
 G  Y  I  D  G  G  A  S  Q  E  E  F  Y  K  F  I  K  P  I  L
gaaaaaatggatggtactgaggaattattggtgaaactaaatcgtgaagatttgctgcgc
 E  K  M  D  G  T  E  E  L  L  V  K  L  N  R  E  D  L  L  R
aagcaacggacctttgacaacggctctattccccatcaaattcacttgggtgagctgcat
 K  Q  R  T  F  D  N  G  S  I  P  H  Q  I  H  L  G  E  L  H
gctattttgagaagacaagaagacttttatccatttttaaaagacaatcgtgagaagatt
 A  I  L  R  R  Q  E  D  F  Y  P  F  L  K  D  N  R  E  K  I
gaaaaaatcttgacttttcgaattccttattatgttggtccattggcgcgtggcaatagt
 E  K  I  L  T  F  R  I  P  Y  Y  V  G  P  L  A  R  G  N  S
cgttttgcatggatgactcggaagtctgaagaaacaattaccccatggaattttgaagaa
 R  F  A  W  M  T  R  K  S  E  E  T  I  T  P  W  N  F  E  E
gttgtcgataaaggtgcttcagctcaatcatttattgaacgcatgacaaactttgataaa
 V  V  D  K  G  A  S  A  Q  S  F  I  E  R  M  T  N  F  D  K
aatcttccaaatgaaaagtactaccaaaacatagtttgctttatgagtattttacggtt
 N  L  P  N  E  K  V  L  P  K  H  S  L  L  Y  E  Y  F  T  V
```

tataacgaattgacaaaggtcaaatatgttactgaaggaatgcgaaaaccagcatttctt
Y N E L T K V K Y V T E G M R K P A F L
tcaggtgaacagaagaaagccattgttgatttactcttcaaaacaaatcgaaaagtaacc
S G E Q K K A I V D L L F K T N R K V T
gttaagcaattaaaagaagattatttcaaaaaaatagaatgttttgatagtgttgaaatt
V K Q L K E D Y F K K I E C F D S V E I
tcaggagttgaagatagatttaatgcttcattaggtacctaccatgatttgctaaaaatt
S G V E D R F N A S L G T Y H D L L K I
attaaagataaagatttttggataatgaagaaaatgaagatatcttagaggatattgtt
I K D K D F L D N E E N E D I L E D I V
ttaacattgaccttatttgaagatagggagatgattgaggaaagacttaaaacatatgct
L T L T L F E D R E M I E E R L K T Y A
cacctctttgatgataaggtgatgaaacagcttaaacgtcgccgttatactggttggga
H L F D D K V M K Q L K R R R Y T G W G
cgtttgtctcgaaaattgattaatggtattagggataagcaatctggcaaaacaatatta
R L S R K L I N G I R D K Q S G K T I L
gatttttgaaatcagatggttttgccaatcgcaatttatgcagctgatccatgatgat
D F L K S D G F A N R N F M Q L I H D D
agtttgacatttaaagaagacattcaaaaagcacaagtgtctggacaaggcgatagttta
S L T F K E D I Q K A Q V S G Q G D S L
catgaacatattgcaaatttagctggtagccctgctattaaaaaaggtattttacagact
H E H I A N L A G S P A I K K G I L Q T
gtaaaagttgttgatgaattggtcaaagtaatggggcggcataagccagaaaatatcgtt
V K V V D E L V K V M G R H K P E N I V
attgaaatggcacgtgaaaatcagacaactcaaaagggccagaaaaattcgcgagagcgt
I E M A R E N Q T T Q K G Q K N S R E R
atgaaacgaatcgaagaaggtatcaaagaattaggaagtcagattcttaaagagcatcct
M K R I E E G I K E L G S Q I L K E H P
gttgaaaatactcaattgcaaaatgaaaagctctatctctattatctccaaaatggaaga
V E N T Q L Q N E K L Y L Y Y L Q N G R
gacatgtatgtggaccaagaattagatattaatcgtttaagtgattatgatgtcgatgcc
D M Y V D Q E L D I N R L S D Y D V D A
attgttccacaaagtttccttaaagacgattcaatagacaataaggtcttaacgcgttct
I V P Q S F L K D D S I D N K V L T R S
gataaaaatcgtggtaaatcggataacgttccaagtgaagaagtagtcaaaaagatgaaa
D K N R G K S D N V P S E E V V K K M K
aactattggagacaacttctaaacgccaagttaatcactcaacgtaagtttgataattta
N Y W R Q L L N A K L I T Q R K F D N L
acgaaagctgaacgtggaggtttgagtgaacttgataaagctggttttatcaaacgccaa
T K A E R G G L S E L D K A G F I K R Q
ttggttgaaactcgccaaatcactaagcatgtggcacaaattttggatagtcgcatgaat
L V E T R Q I T K H V A Q I L D S R M N
actaaatacgatgaaaatgataaacttattcgagaggttaaagtgattaccttaaaatct
T K Y D E N D K L I R E V K V I T L K S
aaattagtttctgacttccgaaaagatttccaattctataaagtacgtgagattaacaat
K L V S D F R K D F Q F Y K V R E I N N
taccatcatgcccatgatgcgtatctaaatgccgtcgttggaactgctttgattaagaaa
Y H H A H D A Y L N A V V G T A L I K K
tatccaaaacttgaatcggagtttgtctatggtgattataaagtttatgatgttcgtaaa
Y P K L E S E F V Y G D Y K V Y D V R K
atgattgctaagtctgagcaagaaataggcaaagcaaccgcaaaatatttcttttactct
M I A K S E Q E I G K A T A K Y F F Y S
aatatcatgaacttcttcaaaacagaaattacacttgcaaatggagagattcgcaaacgc
N I M N F F K T E I T L A N G E I R K R
cctctaatcgaaactaatggggaaactggagaaattgtctgggataaagggcgagatttt
P L I E T N G E T G E I V W D K G R D F
gccacagtgcgcaaagtattgtccatgccccaagtcaatattgtcaagaaaacagaagta
A T V R K V L S M P Q V N I V K K T E V
cagacaggcggattctccaaggagtcaattttaccaaaaagaaattcggacaagcttatt
Q T G G F S K E S I L P K R N S D K L I

```
gctcgtaaaaaagactgggatccaaaaaaatatggtggttttgatagtccaacggtagct
 A  R  K  K  D  W  D  P  K  K  Y  G  G  F  D  S  P  T  V  A
tattcagtcctagtggttgctaaggtggaaaaagggaaatcgaagaagttaaaatccgtt
 Y  S  V  L  V  V  A  K  V  E  K  G  K  S  K  K  L  K  S  V
aaagagttactagggatcacaattatggaaagaagttcctttgaaaaaaatccgattgac
 K  E  L  L  G  I  T  I  M  E  R  S  S  F  E  K  N  P  I  D
tttttagaagctaaaggatataaggaagttaaaaaagacttaatcattaaactacctaaa
 F  L  E  A  K  G  Y  K  E  V  K  K  D  L  I  I  K  L  P  K
tatagtctttttgagttagaaaacggtcgtaaacggatgctggctagtgccggagaatta
 Y  S  L  F  E  L  E  N  G  R  K  R  M  L  A  S  A  G  E  L
caaaaaggaaatgagctggctctgccaagcaaatatgtgaatttttatatttagctagt
 Q  K  G  N  E  L  A  L  P  S  K  Y  V  N  F  L  Y  L  A  S
cattatgaaaagttgaagggtagtccagaagataacgaacaaaaacaattgtttgtggag
 H  Y  E  K  L  K  G  S  P  E  D  N  E  Q  K  Q  L  F  V  E
cagcataagcattatttagatgagattattgagcaaatcagtgaattttctaagcgtgtt
 Q  H  K  H  Y  L  D  E  I  I  E  Q  I  S  E  F  S  K  R  V
attttagcagatgccaatttagataaagttcttagtgcatataacaaacatagagacaaa
 I  L  A  D  A  N  L  D  K  V  L  S  A  Y  N  K  H  R  D  K
ccaatacgtgaacaagcagaaaatattattcatttatttacgttgacgaatcttggagct
 P  I  R  E  Q  A  E  N  I  I  H  L  F  T  L  T  N  L  G  A
cccgctgcttttaaatattttgatacaacaattgatcgtaaacgatatacgtctacaaaa
 P  A  A  F  K  Y  F  D  T  T  I  D  R  K  R  Y  T  S  T  K
gaagttttagatgccactcttatccatcaatccatcactggtctttatgaaacacgcatt
 E  V  L  D  A  T  L  I  H  Q  S  I  T  G  L  Y  E  T  R  I
gatttgagtcagctaggaggtgacaccggtgggggtagcggcggctcgggggggtagtggt
 D  L  S  Q  L  G  G  D  T  G  G  G  S  G  G  S  G  G  S  G
ggaagcggggggttcaaagcttactagtgttaccggctatcgtctgtttgaaaaagagagc
 G  S  G  G  S  K  L  T  S  V  T  G  Y  R  L  F  E  K  E  S
ggatccggtggaagctggagccatccgcagtttgaaaaataaactagtgtcttcacactc
 G  S  G  G  S  W  S  H  P  Q  F  E  K  -  T  S  V  F  T  L
gaagatttcgttggggactgggaacagacagccgcctacaacctggaccaagtccttgaa
 E  D  F  V  G  D  W  E  Q  T  A  A  Y  N  L  D  Q  V  L  E
cagggaggtgtgtccagtttgctgcagaatctcgccgtgtccgtaactccgatccaaagg
 Q  G  G  V  S  S  L  L  Q  N  L  A  V  S  V  T  P  I  Q  R
attgtccggagcggtgaaaatgccctgaagatcgacatccatgtcatcatcccgtatgaa
 I  V  R  S  G  E  N  A  L  K  I  D  I  H  V  I  I  P  Y  E
ggtctgagcgccgaccaaatggcccagatcgaagaggtgtttaaggtggtgtaccctgtg
 G  L  S  A  D  Q  M  A  Q  I  E  E  V  F  K  V  V  Y  P  V
gatgatcatcactttaaggtgatcctgccctatggcacactggtaatcgacggggttacg
 D  D  H  H  F  K  V  I  L  P  Y  G  T  L  V  I  D  G  V  T
ccgaacatgctgaactatttcggacggccgtatgaaggcatcgccgtgttcgacggcaaa
 P  N  M  L  N  Y  F  G  R  P  Y  E  G  I  A  V  F  D  G  K
aagatcactgtaacagggaccctgtggaacggcaacaaaattatcgacgagcgcctgatc
 K  I  T  V  T  G  T  L  W  N  G  N  K  I  I  D  E  R  L  I
acccccgacggctccatgctgttccgagtaaccatcaacagcggtggaagctggagccat
 T  P  D  G  S  M  L  F  R  V  T  I  N  S  G  G  S  W  S  H
ccgcagtttgaaaaataa
 P  Q  F  E  K  -
```

mNG-NL calibrator luciferase

Coding and amino acid sequences for mNeongreen-NanoLuc fusion protein[1,29]. Colour codes: mNeonGreen-ΔC10 in green; NanoLuc-ΔN5 in cyan; His-tag in blue font; StrepTag in red font.

```
atgggcagcagccatcatcatcatcatcacagcagcggcctggtgccgcgcggcagccat
 M  G  S  S  H  H  H  H  H  H  S  S  G  L  V  P  R  G  S  H
atggtaagtaaaggtgaagaagacaatatggcttctctgcctgccacacatgagcttcat
 M  V  S  K  G  E  E  D  N  M  A  S  L  P  A  T  H  E  L  H
attttgggagcataaacggagtggatttcgacatggtaggtcagggtacggggaaccct
 I  F  G  S  I  N  G  V  D  F  D  M  V  G  Q  G  T  G  N  P
aacgatggatatgaggagttgaatcttaaaagcacaaagggtgatctgcagttctcgccc
 N  D  G  Y  E  E  L  N  L  K  S  T  K  G  D  L  Q  F  S  P
tggatcctggtgccgcatataggttatggtttccatcagtatcttccatacccggatggc
 W  I  L  V  P  H  I  G  Y  G  F  H  Q  Y  L  P  Y  P  D  G
atgagcccttttcaggccgcaatggtagatggctcaggatatcaagtgcatcggaccatg
 M  S  P  F  Q  A  A  M  V  D  G  S  G  Y  Q  V  H  R  T  M
cagtttgaagatggggcgtctttgacggtaaattacaggtacacctatgagggtagccat
 Q  F  E  D  G  A  S  L  T  V  N  Y  R  Y  T  Y  E  G  S  H
ataaagggagaagcgcaggtgaagggaactggattcccagcggatggcccagtcatgaca
 I  K  G  E  A  Q  V  K  G  T  G  F  P  A  D  G  P  V  M  T
aacagcctcaccgctgctgattggtgccgatccaagaaaacgtatccaaacgataaaact
 N  S  L  T  A  A  D  W  C  R  S  K  K  T  Y  P  N  D  K  T
atcatttctacttttaagtggtcctatacaacaggaaacggaaacgctatcgttcaacg
 I  I  S  T  F  K  W  S  Y  T  T  G  N  G  K  R  Y  R  S  T
gcccgcacgacctacacgtttgcaaagccaatggctgcgaattatctgaaaaaccagccg
 A  R  T  T  Y  T  F  A  K  P  M  A  A  N  Y  L  K  N  Q  P
atgtatgtgttccgtaaaaccgaactgaaacattctaaaacggagctcaatttcaaggaa
 M  Y  V  F  R  K  T  E  L  K  H  S  K  T  E  L  N  F  K  E
tggcagaaggcatttaccggttttgaagatttcgtgggtgattggcgacaaacggccggt
 W  Q  K  A  F  T  G  F  E  D  F  V  G  D  W  R  Q  T  A  G
tacaatttggatcaggtgttagaacaagggggcgtaagctccctgttccagaatttagga
 Y  N  L  D  Q  V  L  E  Q  G  G  V  S  S  L  F  Q  N  L  G
gtgagcgtgacacctattcagcgcattgtgctgagcggcgaaaatggtctgaaaattgat
 V  S  V  T  P  I  Q  R  I  V  L  S  G  E  N  G  L  K  I  D
attcatgtgatcatcccttacgaaggcctgtctggggatcaaatgggacagattgaaaaa
 I  H  V  I  I  P  Y  E  G  L  S  G  D  Q  M  G  Q  I  E  K
atcttcaaagtagtttatccggtcgacgatcatcattttaaagtaattctgcactatggg
 I  F  K  V  V  Y  P  V  D  D  H  H  F  K  V  I  L  H  Y  G
acactcgttatcgatggagtcacgccgaatatgatagactacttcggtcgcccgtacgaa
 T  L  V  I  D  G  V  T  P  N  M  I  D  Y  F  G  R  P  Y  E
ggaatcgcggttttcgatggaaaaaaaatcacagtaacgggcacattgtggaacgggaat
 G  I  A  V  F  D  G  K  K  I  T  V  T  G  T  L  W  N  G  N
aaaatcatagacgaacgcctcattaaccctgatggatctttactgttccgcgtcacaatt
 K  I  I  D  E  R  L  I  N  P  D  G  S  L  L  F  R  V  T  I
aatggcgttacaggttggcgactgtgtgaacgtattctcgcaggtaccacatctgcgtgg
 N  G  V  T  G  W  R  L  C  E  R  I  L  A  G  T  T  S  A  W
agccatcctcagttcgaaaaataa
 S  H  P  Q  F  E  K  -
```

References

(1) Ni, Y.; Rosier, B. J. H. M.; van Aalen, E. A.; Hanckmann, E. T. L.; Biewenga, L.; Pistikou, A. M. M.; Timmermans, B.; Vu, C.; Roos, S.; Arts, R.; Li, W.; de Greef, T. F. A.; van Borren, M. M. G. J.; van Kuppeveld, F. J. M.; Bosch, B. J.; Merkx, M. A Plug-and-Play Platform of Ratiometric Bioluminescent Sensors for Homogeneous Immunoassays. *Nat Commun* **2021**, *12*, 4586. https://doi.org/10.1038/s41467-021-24874-3.

(2) Haeussler, M.; Schönig, K.; Eckert, H.; Eschstruth, A.; Mianné, J.; Renaud, J. B.; Schneider-Maunoury, S.; Shkumatava, A.; Teboul, L.; Kent, J.; Joly, J. S.; Concordet, J. P. Evaluation of Off-Target and on-Target Scoring Algorithms and Integration into the Guide RNA Selection Tool CRISPOR. *Genome Biol* **2016**, *17*, 148. https://doi.org/10.1186/s13059-016-1012-2.

(3) Concordet, J. P.; Haeussler, M. CRISPOR: Intuitive Guide Selection for CRISPR/Cas9 Genome Editing Experiments and Screens. *Nucleic Acids Res* **2018**, *46* (W1), W242–W245. https://doi.org/10.1093/nar/gky354.

(4) Moreno-Mateos, M. A.; Vejnar, C. E.; Beaudoin, J. D.; Fernandez, J. P.; Mis, E. K.; Khokha, M. K.; Giraldez, A. J. CRISPRscan: Designing Highly Efficient SgRNAs for CRISPR-Cas9 Targeting in Vivo. *Nat Methods* **2015**, *12* (10), 982–988. https://doi.org/10.1038/nmeth.3543.

(5) Doench, J. G.; Fusi, N.; Sullender, M.; Hegde, M.; Vaimberg, E. W.; Donovan, K. F.; Smith, I.; Tothova, Z.; Wilen, C.; Orchard, R.; Virgin, H. W.; Listgarten, J.; Root, D. E. Optimized SgRNA Design to Maximize Activity and Minimize Off-Target Effects of CRISPR-Cas9. *Nat Biotechnol* **2016**, *34* (2), 184–191. https://doi.org/10.1038/nbt.3437.

(6) Hsu, P. D.; Scott, D. A.; Weinstein, J. A.; Ran, F. A.; Konermann, S.; Agarwala, V.; Li, Y.; Fine, E. J.; Wu, X.; Shalem, O.; Cradick, T. J.; Marraffini, L. A.; Bao, G.; Zhang, F. DNA Targeting Specificity of RNA-Guided Cas9 Nucleases. *Nat Biotechnol* **2013**, *31* (9), 827–832. https://doi.org/10.1038/nbt.2647.

(7) Higgins, M.; Ravenhall, M.; Ward, D.; Phelan, J.; Ibrahim, A.; Forrest, M. S.; Clark, T. G.; Campino, S. PrimedRPA: Primer Design for Recombinase Polymerase Amplification Assays. *Bioinformatics* **2019**, *35* (4), 682–684. https://doi.org/10.1093/bioinformatics/bty701.

(8) Shu, Y.; McCauley, J. GISAID: Global Initiative on Sharing All Influenza Data – from Vision to Reality. *Eurosurveillance* **2017**, *22* (13), 3049. https://doi.org/10.2807/1560-7917.ES.2017.22.13.30494.

(9) Hadfield, J.; Megill, C.; Bell, S. M.; Huddleston, J.; Potter, B.; Callender, C.; Sagulenko, P.; Bedford, T.; Neher, R. A. NextStrain: Real-Time Tracking of Pathogen Evolution. *Bioinformatics* **2018**, *34* (23), 4121–4123. https://doi.org/10.1093/bioinformatics/bty407.

(10) Fernandes, J. D.; Hinrichs, A. S.; Clawson, H.; Gonzalez, J. N.; Lee, B. T.; Nassar, L. R.; Raney, B. J.; Rosenbloom, K. R.; Nerli, S.; Rao, A. A.; Schmelter, D.; Fyfe, A.; Maulding, N.; Zweig, A. S.; Lowe, T. M.; Ares, M.; Corbet-Detig, R.; Kent, W. J.; Haussler, D.; Haeussler, M. The UCSC SARS-CoV-2 Genome Browser. *Nat Genet* **2020**, *52* (10), 991–998. https://doi.org/10.1038/s41588-020-0700-8.

(11) Shrivastava, A.; Gupta, V. Methods for the Determination of Limit of Detection and Limit of Quantitation of the Analytical Methods. *Chronicles of Young Scientists* **2011**, *2* (1), 21–25. https://doi.org/10.4103/2229-5186.79345.

(12) Arizti-Sanz, J.; Freije, C. A.; Stanton, A. C.; Petros, B. A.; Boehm, C. K.; Siddiqui, S.; Shaw, B. M.; Adams, G.; Kosoko-Thoroddsen, T. S. F.; Kemball, M. E.; Uwanibe, J. N.; Ajogbasile, F. v.; Eromon, P. E.; Gross, R.; Wronka, L.; Caviness, K.; Hensley, L. E.; Bergman, N. H.; MacInnis, B. L.; Happi, C. T.; Lemieux, J. E.; Sabeti, P. C.; Myhrvold, C. Streamlined Inactivation, Amplification, and Cas13-Based Detection of SARS-CoV-2. *Nat Commun* **2020**, *11* (1), 5921. https://doi.org/10.1038/s41467-020-19097-x.

(13) Qian, J.; Boswell, S. A.; Chidley, C.; Lu, Z. xiang; Pettit, M. E.; Gaudio, B. L.; Fajnzylber, J. M.; Ingram, R. T.; Ward, R. H.; Li, J. Z.; Springer, M. An Enhanced Isothermal Amplification Assay for Viral Detection. *Nat Commun* **2020**, *11*, 5920. https://doi.org/10.1038/s41467-020-19258-y.

(14) Motohashi, K. A Simple and Fast Manual Centrifuge to Spin Solutions in 96-Well Pcr Plates. *Methods Protoc* **2020**, *3*, 41. https://doi.org/10.3390/mps3020041.

(15) Gong, S.; Yu, H. H.; Johnson, K. A.; Taylor, D. W. DNA Unwinding Is the Primary Determinant of CRISPR-Cas9 Activity. *Cell Rep* **2018**, *22* (2), 359–371. https://doi.org/10.1016/j.celrep.2017.12.041.

(16) Liu, M. sen; Gong, S.; Yu, H. H.; Jung, K.; Johnson, K. A.; Taylor, D. W. Engineered CRISPR/Cas9 Enzymes Improve Discrimination by Slowing DNA Cleavage to Allow Release of off-Target DNA. *Nat Commun* **2020**, *11* (1), 3576. https://doi.org/10.1038/s41467-020-17411-1.

(17) Geertjens, N. H. J.; de Vink, P. J.; Wezeman, T.; Markvoort, A. J.; Brunsveld, L. A General Framework for Straightforward Model Construction of Multi-Component Thermodynamic Equilibrium Systems. *bioRxiv* **2021**. https://doi.org/10.1101/2021.11.18.469126.

(18) Dixon, A. S.; Schwinn, M. K.; Hall, M. P.; Zimmerman, K.; Otto, P.; Lubben, T. H.; Butler, B. L.; Binkowski, B. F.; MacHleidt, T.; Kirkland, T. A.; Wood, M. G.; Eggers, C. T.; Encell, L. P.; Wood, K. v. NanoLuc Complementation Reporter Optimized for Accurate Measurement of Protein Interactions in Cells. *ACS Chem Biol* **2016**, *11* (2), 400–408. https://doi.org/10.1021/acschembio.5b00753.

(19) Sternberg, S. H.; Redding, S.; Jinek, M.; Greene, E. C.; Doudna, J. A. DNA Interrogation by the CRISPR RNA-Guided Endonuclease Cas9. *Nature* **2014**, *507* (7490), 62–67. https://doi.org/10.1038/nature13011.

(20) Kjaergaard, M.; Glavina, J.; Chemes, L. B. Predicting the Effect of Disordered Linkers on Effective Concentrations and Avidity with the “Ceff Calculator” App. In *Methods in Enzymology*; Merkx, M., Ed.; Academic Press Inc., **2021**; Vol. 647, pp 145–171. https://doi.org/10.1016/bs.mie.2020.09.012.

(21) Ceballos-Alcantarilla, E.; Merkx, M. Understanding and Applications of Ser/Gly Linkers in Protein Engineering. In *Methods in Enzymology*; Merkx, M., Ed.; Academic Press Inc., **2021**; Vol. 647, pp 1–22. https://doi.org/10.1016/bs.mie.2020.12.001.

(22) Jiang, F.; Taylor, D. W.; Chen, J. S.; Kornfeld, J. E.; Zhou, K.; Thompson, A. J.; Nogales, E.; Doudna, J. A. Structures of a CRISPR-Cas9 R-Loop Complex Primed for DNA Cleavage. *Science* **2016**, *351* (6275), 867–871. https://doi.org/10.1126/science.aad8282.

(23) Arizti-Sanz, J.; Bradley, A.; Zhang, Y. B.; Boehm, C. K.; Freije, C. A.; Grunberg, M. E.; Kosoko-Thoroddsen, T.-S. F.; Welch, N. L.; Pillai, P. P.; Mantena, S.; Kim, G.; Uwanibe, J. N.; John, O. G.; Eromon, P. E.; Kocher, G.; Gross, R.; Lee, J. S.; Hensley, L. E.; MacInnis, B. L.; Johnson, J.; Springer, M.; Happi, C. T.; Sabeti, P. C.; Myhrvold, C. Simplified Cas13-Based Assays for the Fast Identification of SARS-CoV-2 and Its Variants. *Nat Biomed Eng* **2022**, *6*, 932–943. https://doi.org/10.1038/s41551-022-00889-z.

(24) Broughton, J. P.; Deng, X.; Yu, G.; Fasching, C. L.; Servellita, V.; Singh, J.; Miao, X.; Streithorst, J. A.; Granados, A.; Sotomayor-gonzalez, A.; Zorn, K.; Gopez, A.; Hsu, E.; Gu, W.; Miller, S.; Pan, C. Y.; Guevara, H.; Wadford, D. A.; Chen, J. S.; Chiu, C. Y. CRISPR–Cas12-Based Detection of SARS-CoV-2. *Nat Biotechnol* **2020**, *38*, 870–874. https://doi.org/10.1038/s41587-020-0513-4.
(25) Steens, J. A.; Zhu, Y.; Taylor, D. W.; Bravo, J. P. K.; Prinsen, S. H. P.; Schoen, C. D.; Keijser, B. J. F.; Ossendrijver, M.; Hofstra, L. M.; Brouns, S. J. J.; Shinkai, A.; van der Oost, J.; Staals, R. H. J. SCOPE Enables Type III CRISPR-Cas Diagnostics Using Flexible Targeting and Stringent CARF Ribonuclease Activation. *Nat Commun* **2021**, *12* (1), 5033. https://doi.org/10.1038/s41467-021-25337-5.
(26) Joung, J.; Ladha, A.; Saito, M.; Kim, N.-G.; Woolley, A. E.; Segel, M.; Barretto, R. P. J.; Ranu, A.; Macrae, R. K.; Faure, G.; Ioannidi, E. I.; Krajeski, R. N.; Bruneau, R.; Huang, M.-L. W.; Yu, X. G.; Li, J. Z.; Walker, B. D.; Hung, D. T.; Greninger, A. L.; Jerome, K. R.; Gootenberg, J. S.; Abudayyeh, O. O.; Zhang, F. Detection of SARS-CoV-2 with SHERLOCK One-Pot Testing. *New England Journal of Medicine* **2020**, *383* (15), 1492–1494. https://doi.org/10.1056/nejmc2026172.
(27) Chandrasekaran, S. S.; Agrawal, S.; Fanton, A.; Jangid, A. R.; Charrez, B.; Escajeda, A. M.; Son, S.; Mcintosh, R.; Tran, H.; Bhuiya, A.; de León Derby, M. D.; Switz, N. A.; Armstrong, M.; Harris, A. R.; Prywes, N.; Lukarska, M.; Biering, S. B.; Smock, D. C. J.; Mok, A.; Knott, G. J.; Dang, Q.; van Dis, E.; Dugan, E.; Kim, S.; Liu, T. Y.; IGI Testing Consortium; Moehle, E. A.; Kogut, K.; Eskenazi, B.; Harris, E.; Stanley, S. A.; Lareau, L. F.; Tan, M. X.; Fletcher, D. A.; Doudna, J. A.; Savage, D. F.; Hsu, P. D. Rapid Detection of SARS-CoV-2 RNA in Saliva via Cas13. *Nat Biomed Eng* **2022**, *6*, 944–956. https://doi.org/10.1038/s41551-022-00917-y.
(28) Lu, S.; Tong, X.; Han, Y.; Zhang, K.; Zhang, Y.; Chen, Q.; Duan, J.; Lei, X.; Huang, M.; Qiu, Y.; Zhang, D. Y.; Zhou, X.; Zhang, Y.; Yin, H. Fast and Sensitive Detection of SARS-CoV-2 RNA Using Suboptimal Protospacer Adjacent Motifs for Cas12a. *Nat Biomed Eng* **2022**, *6* (3), 286–297. https://doi.org/10.1038/s41551-022-00861-x.
(29) Suzuki, K.; Kimura, T.; Shinoda, H.; Bai, G.; Daniels, M. J.; Arai, Y.; Nakano, M.; Nagai, T. Five Colour Variants of Bright Luminescent Protein for Real-Time Multicolour Bioimaging. *Nat Commun* **2016**, *7*, 13718. https://doi.org/10.1038/ncomms13718.
